# Potentiating dual-directional immunometabolic regulation with nanomedicine to enhance anti-tumor immunotherapy following incomplete photothermal ablation

**DOI:** 10.1186/s12951-024-02643-w

**Published:** 2024-06-24

**Authors:** Qinqin Jiang, Bin Qiao, Jun Zheng, Weixiang Song, Nan Zhang, Jie Xu, Jia Liu, Yixin Zhong, Qin Zhang, Weiwei Liu, Lanlan You, Nianhong Wu, Yun Liu, Pan Li, Haitao Ran, Zhigang Wang, Dajing Guo

**Affiliations:** 1 Department of Ultrasound, Chongqing Key Laboratory of Ultrasound Molecular Imaging, Second Affiliated Hospital of Chongqing Medical University, Chongqing, 400010 P. R. China; 2 Department of Radiology, the Second Affiliated Hospital of Chongqing Medical University, Chongqing, 400010 P. R. China; 3 Department of Medical Ultrasonics, the First Affiliated Hospital of Sun Yat-sen University, Guangzhou, 510080 P. R. China; 4 Department of Radiology, Chongqing Hospital of Traditional Chinese Medicine, Chongqing, 400021 P. R. China; 5 Department of Ultrasound, Clinical Medical College and the First Affiliated Hospital of Chengdu Medical College, Chengdu, 610500 P. R. China

**Keywords:** Photothermal therapy, Adenosine metabolism, Immune checkpoint blockade, Immunosuppressive microenvironment, Biomaterials

## Abstract

Photothermal therapy (PTT) is a promising cancer treatment method due to its ability to induce tumor-specific T cell responses and enhance therapeutic outcomes. However, incomplete PTT can leave residual tumors that often lead to new metastases and decreased patient survival in clinical scenarios. This is primarily due to the release of ATP, a damage-associated molecular pattern that quickly transforms into the immunosuppressive metabolite adenosine by CD39, prevalent in the tumor microenvironment, thus promoting tumor immune evasion. This study presents a photothermal nanomedicine fabricated by electrostatic adsorption among the Fe-doped polydiaminopyridine (Fe-PDAP), indocyanine green (ICG), and CD39 inhibitor sodium polyoxotungstate (POM-1). The constructed Fe-PDAP@ICG@POM-1 (FIP) can induce tumor PTT and immunogenic cell death when exposed to a near-infrared laser. Significantly, it can inhibit the ATP-adenosine pathway by dual-directional immunometabolic regulation, resulting in increased ATP levels and decreased adenosine synthesis, which ultimately reverses the immunosuppressive microenvironment and increases the susceptibility of immune checkpoint blockade (aPD-1) therapy. With the aid of aPD-1, the dual-directional immunometabolic regulation strategy mediated by FIP can effectively suppress/eradicate primary and distant tumors and evoke long-term solid immunological memory. This study presents an immunometabolic control strategy to offer a salvage option for treating residual tumors following incomplete PTT.

**Supplementary Information:**

The online version contains supplementary material available at 10.1186/s12951-024-02643-w.

## Introduction

Photothermal therapy (PTT) is an effective curative thermal ablation strategy with high spatiotemporal precision, non-invasion, and low toxicity [1–4]. However, most PTT cannot undergo complete ablation, which limits its efficacy and further clinical applications [5]. Incomplete thermal ablation resulting from an ill-defined tumor boundary or undetected micrometastases provides the foundation for tumor recurrence [6–9]. Photothermal transduction agents (PTAs) transform light energy into heat and then raise the ambient temperature at the tumor location, resulting in more effective tumor cell killing [10, 11]. Despite its broad prospects, it is still challenging to eliminate tumor cells using PTAs-assisted PTT alone [12–14]. Besides, the total eradication of localized tumors requires high ablation power and extensive ablation duration, leading to biosafety concerns due to the elevated temperature [15, 16]. Given this predicament, it is imperative to develop new methods promptly to enhance the long-term outcomes of PTT.

Immune checkpoint blockade(ICB)therapy has shown promise in cancer treatment but exhibits limited success in solid tumors [17, 18], particularly those with incomplete thermal ablation, such as radiofrequency ablation [19]. The failure in tumor therapy highlights the need for a more comprehensive knowledge of the molecular processes that suppress immune responses within the challenging tumor microenvironment after incomplete thermal ablation. Although PTT can facilitate the release of tumor antigens and promote immunogenic cell death (ICD) [20, 21], the benefits are often undermined by an immunosuppressive environment, mainly due to the accumulation of myeloid cells like myeloid-derived suppressor cells (MDSCs) and tumor-associated macrophages (TAMs). Moreover, incomplete radiofrequency ablation has been linked to malignant progression, increased tumor cell proliferation, and enhanced endothelial permeability [22, 23]. Shi et colleagues found that incomplete radiofrequency ablation can mediate local recruitment of monocytes and TAMs to reduce immune surveillance and hamper aPD-1 efficacy [19]. Efforts to uncover and repress the fundamental principles that cause these persistent tumors, which have pro-oncogenic and immunosuppressive effects, are urgently needed.

PTT can induce ICD and release adenosine-5-triphosphate (ATP), a prevalent damage-associated molecular pattern [24, 25]. ATP acts as a “find-me” signal, facilitating the display of antigens to stimulate T cells and initiate immunological responses against cancer. Unfortunately, tumors have evolved an effective strategy to diminish this benefit via the ATP-adenosine (Ade) axis [26, 27]. ATP can be degraded by CD39, which is widely expressed in the TME and results in the formation of AMP [26]. Subsequently, CD73 in the TME phosphorylates AMP and converts it into immunosuppressive metabolite Ade [26]. The excessive production of Ade can not only eradicate the immune activation response induced by ICD but also create an extremely immune-suppressive milieu that facilitates the excessive development of tumors and severely damages aPD-1 efficacy [28]. Regulating crucial stages in the ATP-Ade pathway has promise for altering the immunosuppressive TME and enhancing the clinical results of photothermal immunotherapy [29, 30]. Of all the options, CD39 inhibitors have the highest potential for improving the treatment outcome [31, 32]. It can prevent immunological suppression induced by adenosine anabolism, limit ATP breakdown, and boost ICD effects to achieve dual-directional immunoregulation. However, the extensive distribution of CD39 inhibitors in tissues can non-specifically inhibit the ATP-Ade pathway and unavoidably result in systematic adverse effects. Therefore, targeting the CD39-mediated ATP-Ade pathway with the dual-directional immunometabolic nanomedicine may provide new strategies to treat tumors that undergo incomplete PTT.

**Scheme 1:**
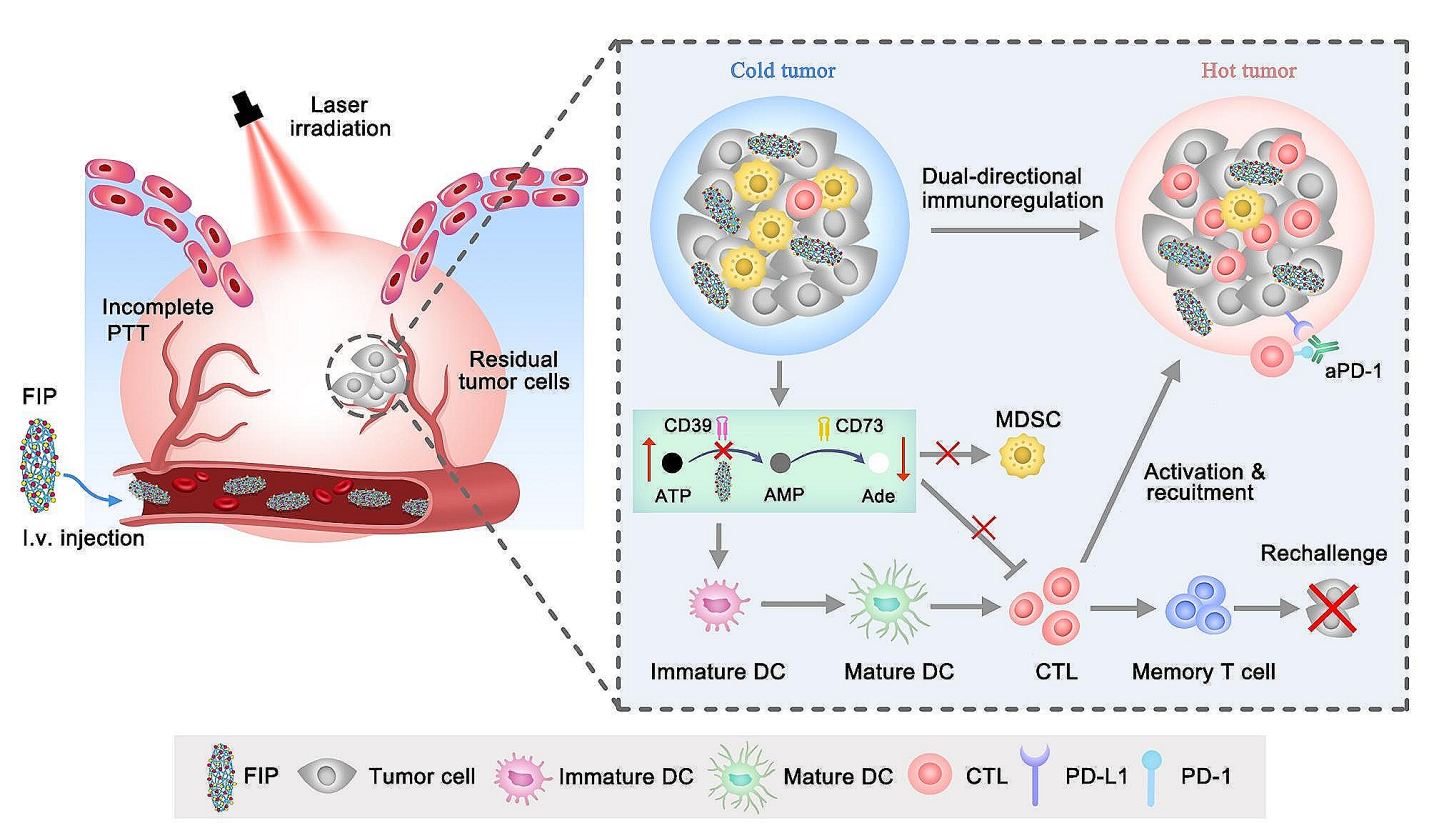
Schematic illustration of dual-directional immunometabolic regulation strategy in combination with aPD-1 to provide effective cancer treatment after incomplete PTT.

In this study, for the first time, we found that the CD39-mediated ATP-Ade pathway activated by incomplete PTT promoted the expansion of myeloid cell populations and deteriorated immunosuppressive milieu, ultimately promoting the over-development of residual tumors. Inspired by this discovery, we introduced CD39 inhibitor sodium polyoxotungstate (POM-1) and developed an immunometabolic regulator Fe-PDAP@ICG@POM-1 (FIP) nanoparticles (NPs), composed of Fe-PDAP (Fe-doped polydiaminopyridine) NPs, loaded with the photothermal agent indocyanine green (ICG) and POM-1.ICG exhibits enhanced photothermal conversion efficiency to achieve PTT against tumors. By inhibiting CD39, POM-1 can limit ATP breakdown and inhibit Ade production, achieving dual-directional immunometabolic regulation, and enhancing the immune response by increasd ATP level and mitigating the immunosuppressive microenvironment by reduced Ade level. The dual-directional immunometabolic regulation strategy mediated by FIP can enhance anti-tumor immunotherapy after incomplete PTT. As shown in Scheme 1, the combination of PTT with Ade generation and ATP decomposition dual-inhibition can effectively transform the immunosuppressive TME into an immunogenic state. This transformation can significantly enhance the vulnerability of aPD-1 immunotherapy. As expected, the combination of FIP with aPD-1 inhibition showed a superior suppression effect on both primary and distant metastatic tumors and remarkably avoided tumor recurrence. Overall, in this work, we proposed a dual-directional immunometabolic regulation strategy in preventing cancer recurrence and metastasis following PTT and highlighted the potential of manipulating the immunosuppressive TME using immunometabolic regulating nanosystems to enhance the effectiveness of PTT-immunotherapy.

## Results and discussion

### Characterization of FI

**Fig. 1:**
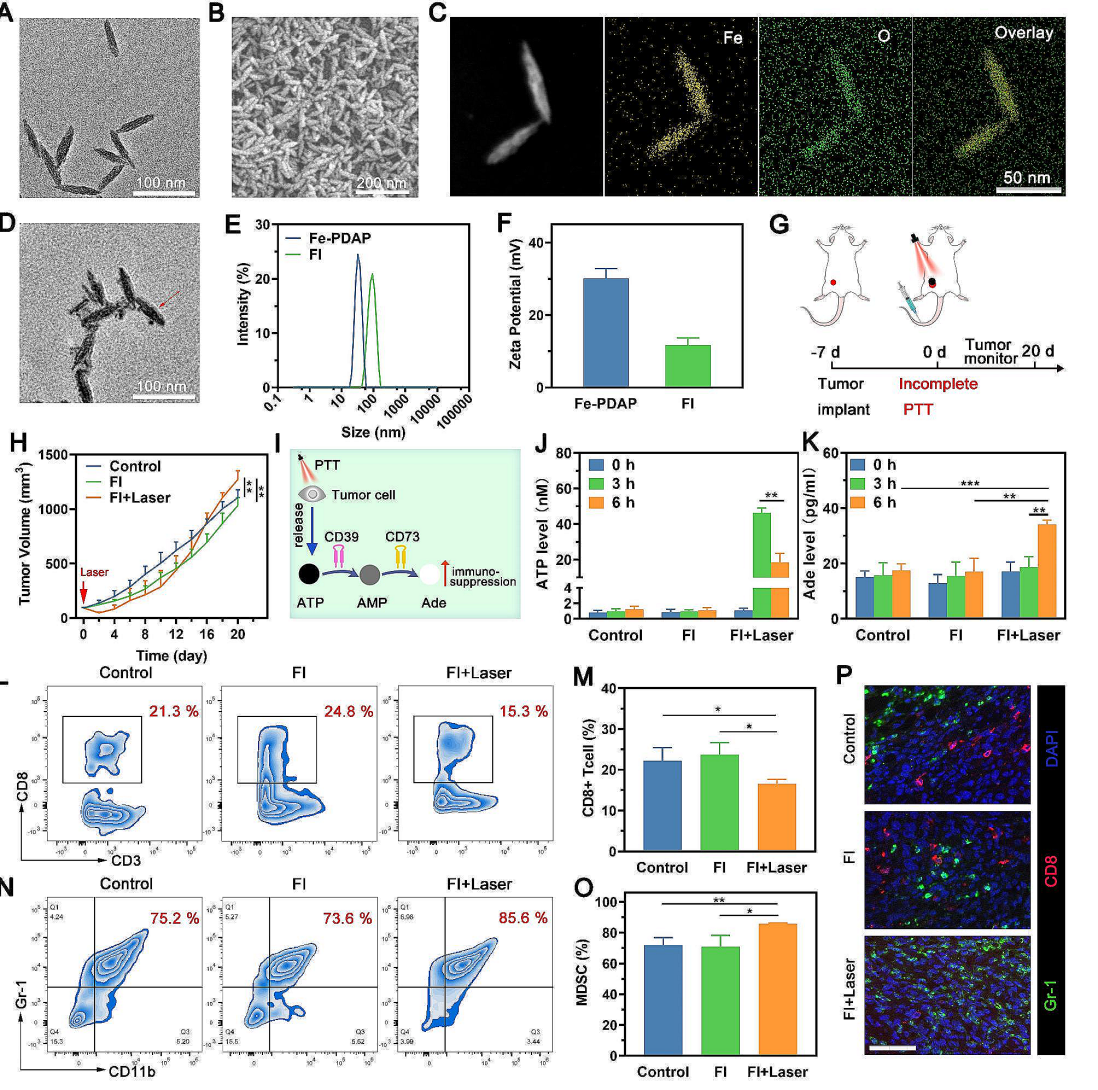
FI characterization and increased accumulation of Ade after incomplete PTT exacerbate the immunosuppressive microenvironment. (**A**-**B**) The TEM and SEM images of Fe-PDAP. (**C**) Elemental distribution mappings of Fe-PDAP. (**D**) TEM image of FI. (**E**) DLS results of Fe-PDAP and FI. (**F**) Zeta potentials of Fe-PDAP and FI. (**G**) Schematic illustration of incomplete PTT. (**H**) Tumor growth curves after different treatments. (**I**) Schematic illustration of Ade production. (**J**-**K**) ATP and Ade levels in 4T1-cell culture medium after different treatments. (**L**) Sample flow cytometry plots displaying CD8 + T cells and (**M**) associated quantitative data following various treatments. (**N**) Sample flow cytometry plots displaying MDSC cells and (**O**) associated quantitative data following differentmultiple treatments. (**P**) Representative polychromatic immunofluorescent staining images of tumors showing DAPI (blue), CD8+ (red) and Gr-1+ (green) cells infiltration in different groups. Scale bar: 50 μm

Based on our previously reported method, Fe-PDAP NPs were created using a simple aqueous polymerization technique to load ICG [33]. Transmission electron microscopy (TEM) and scanning electron microscopy (SEM) results showed that the morphology of Fe-PDAP NPs is highly uniform (Fig. 1A and B). The presence of Fe and O in the Fe-PDAP NPs was verified by element mapping analysis (Fig. 1C). Fe-PDAP NPs were loaded with ICG to create Fe-PDAP@ICG (FI) NPs. TEM result revealed a halo ring on the nanoparticles, confirming the existence of free ICG loading onto the Fe-PDAP (Fig. 1D). When observed by SEM, the morphology of FI did not alter when compared to naked Fe-PDAP (Additional file 1: Figure S1). The size of Fe-PDAP increased to approximately 92 nm after adding ICG (Fig. 1E). The Fe-PDAP potential was measured to be + 30.1 mV, which was reduced to + 11.7 mV after adding ICG (Fig. 1F), further confirming the successful loading of ICG. Furthermore, UV-vis-near-infrared (NIR) spectra were used to demonstrate the adsorption of ICG onto the Fe-PDAP (Additional file 1: Figure S2). The UV-Vis absorption spectrum of ICG exhibited absorption peaks at both 713 nm and 776 nm. Moreover, FI showed similar adsorption peaks at 738 nm and 817 nm, which were ascribed to the red-shift phenomenon due to the formation of J-aggregates and efficient loading of ICG [34, 35]. The loading efficiency of ICG into FI was quantified to be around 94% using the ICG standard curve (Additional file 1: Figure. S3). The successful loading of ICG was further validated by the color disparity between Fe-PDAP and FI (Additional file 1: Figure S4).

### Increased generation of Ade after incomplete PTT deteriorates immunosuppressive microenvironment

We first produced a typical photothermal nanomedicine, FI, and then investigated the potential correlation between the existence of residual tumors and accelerated tumor aggression following FI mediated incomplete PTT on a 4T1 breast cancer model (Fig. 1G). Our findings indicated that incomplete PTT initially decreased the dimensions of the treated tumor (Fig. 1H). Nevertheless, the remaining tumors ultimately exhibited a more significant increase in size compared to the control group and FI group, and the group treated with FI alone had no discernible difference compared to the control group. Significantly, there was an increased incidence of metastasis in the lung on the 20th day following incomplete PTT (Additional file 1: Figure S5); the outcome was consistent with clinical data that residual tumors become more aggressive than untreated tumors across cancers following incomplete thermal ablation, such as radiofrequency ablation [7, 36]. Together, these findings indicated that the existence of the remaining tumor led to fast tumor progression following incomplete PTT.

Ade plays a significant role in the development of tumor immune suppression. Subsequently, we acquired residual 4T1 tumors and investigated alteration in intrinsic characteristics of the TME after incomplete PTT. The result showed that the Ade level of the FI + Laser group was significantly elevated compared with that in other groups (Additional file 1: Figure S6). To further explore the underlying mechanism of Ade production, we performed ATP and Ade analysis in vitro based on the conversion relationship between ATP and Ade (Fig. 1I). As shown in Fig. 1J, the FI + Laser group showed considerably elevated ATP at 3 h, indicating PTT could release ATP through ICD. Specifically, a significant decrease of ATP was found at 6 h in the FI + Laser group compared to the ATP level of the FI + Laser group at 3 h. On the other hand, we observed a significant increase of Ade in the FI + Laser group at 6 h, compared to other groups at 6 h (Fig. 1K). The above results can be attributed to PTT-induced ATP release and ATP could be degraded into immunosuppressive metabolite Ade.

Immunosuppressive Ade can promote the proliferation of protumorigenic immune suppressor MDSCs and hinder the proliferation of antitumorigenic immune effector cells, such as CD8 + T cells [37]. Therefore, we analyzed the infiltration of immune cells in residual 4T1 tumors and compared them to the immune cells present in untreated tumors and the group treated solely with FI alone. Compared to other groups, the FI + Laser group showed a significant decrease in the percentage of CD8 + T cells in the residual tumor (Fig. 1L and M). Additionally, the FI + Laser group exhibited a pronounced immune suppression, which was identified in MDSC cells (Fig. 1N and O). Concurrently, the FI + Laser group showed decreased CD8 + T cells and increased MDSCs in the remaining tumors, as seen by the polychromatic immunofluorescent staining figures (Fig. 1P). These findings indicated that incomplete PTT led to a highly immune-suppressive environment in remaining tumors because of the increased generation of Ade, which promoted MDSCs generation and inhibited CD8 + T cell proliferation. Therefore, it is reasonable to consider that reducing Ade inside the TME might be a viable approach to alter the immunosuppressive microenvironment following incomplete PTT.

### Characterization of dual-directional immunometabolic regulator FIP

**Fig. 2:**
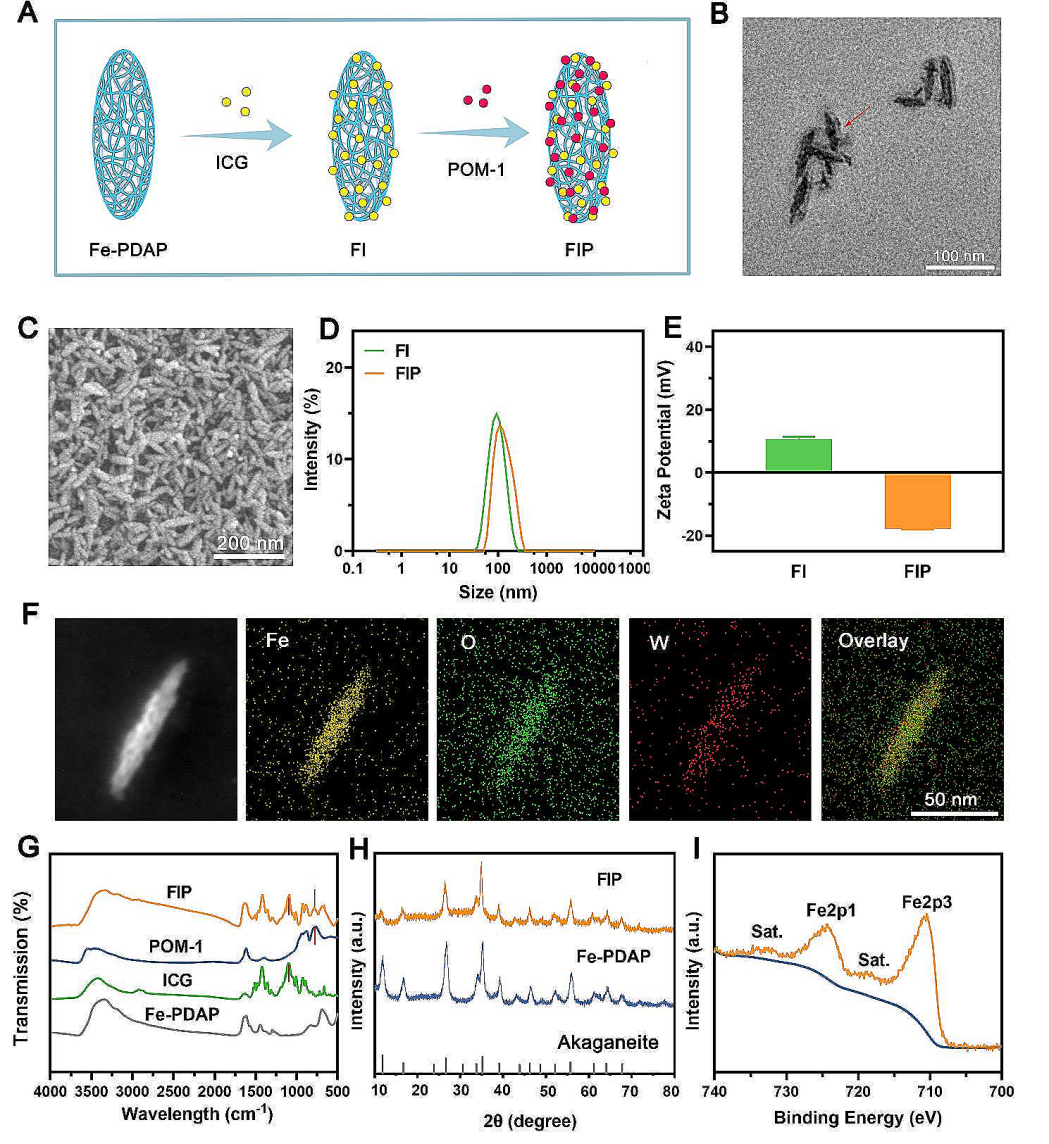
Characterization of FIP. (**A**) Diagram depicting the synthetic methods used to create engineered FIP. (**B**) TEM image of FIP. (**C**) SEM image of FIP. (**D**) Size distribution of FI and FIP. (**E**) Zeta potentials of FI and FIP. (**F**) Elemental mappings image of FIP. (**G**) FTIR spectra of Fe-PDAP, ICG, POM-1 and FIP. (**H**) XRD result of FIP and Fe-PDAP. (**I**) Fe2p spectrum of FIP captured using XPS at a high resolution

Regulating crucial stages in the ATP-Ade pathway has promise for altering the Ade-driven immunosuppressive TME following incomplete PTT. As CD39 occupies an apical and rate-limiting step in the ATP breakdown and Ade generation process, CD39 inhibitors have the highest potential for enhancing the treatment outcome. Based on this, a dual-directional immunometabolic regulator FIP was developed to increase ATP level and minimize the generation of Ade following incomplete PTT. We included the photothermal agent ICG and the CD39 inhibitor POM-1 to regulate Ade metabolism during PTT and enhance the immune response (Fig. 2A). When observed using TEM, the FIP displayed a circular ring around the Fe-PDAP NPs, indicating the presence of ICG and/or POM-1 (Fig. 2B). Upon examination using both TEM and SEM, it was revealed that the morphology of FIP mainly remained unchanged compared to FI (Fig. 2C); this suggested that the encapsulation process maintained the fundamental morphology of Fe-PDAP. The dynamic light scattering (DLS) result indicated that the size of FI was around 92 nm and then rose to 108 nm following the loading of POM-1 (Fig. 2D). This result suggested the successful loading of POM-1. The primary determinant for adopting ICG and POM-1 was the electrostatic attraction between positive and negative charges. The zeta potential of FI was measured to be + 10.68 mV and reverted to -17.85 mV with the addition of POM-1 (Fig. 2E). The loading efficiency of ICG onto Fe-PDAP NPs was calculated to be 89% using UV-vis-NIR spectra analysis (Additional file 1: Figure S7). Conversely, the loading efficiency of POM-1 was found to be 62% using inductively coupled plasma optical mass spectrometry (ICP-MS). Elemental mapping analysis subsequently verified the presence of iron (Fe), oxygen (O), and tungsten (W) in the FIP, further indicating the successful loading of POM-1 (Fig. 2F). The Fourier-transform infrared (FTIR) spectrum of FIP showed characteristic absorption peaks of POM-1 at 777 cm^− 1^, providing further evidence of the existence of POM-1 molecules (Fig. 2G). Furthermore, the FTIR spectra of FIP exhibited distinct absorption peaks at 1091 cm^− 1^, confirming ICG molecules’ existence. Notably, the powder X-ray diffraction (PXRD) patterns of FIP and Fe-PDAP showed no detectable changes, indicating that the manufacturing method did not alter the physical structure of Fe-PDAP (Fig. 2H). According to the standard XRD card, the peak of Fe-PDAP was recognized with akaganeite. Furthermore, the X-ray photoelectron spectroscopy (XPS) spectra demonstrated the durability of Fe (III) in FIP, as evidenced by the characteristic binding energy peaks of Fe 2p1/2 and Fe 2p3/2 (Fig. 2I, Additional file 1: Figure S8). The results indicated the practical fabrication of FIP for further investigations.

### In *vitro* photothermal property and changes in ATP/Ade concentration

**Fig. 3:**
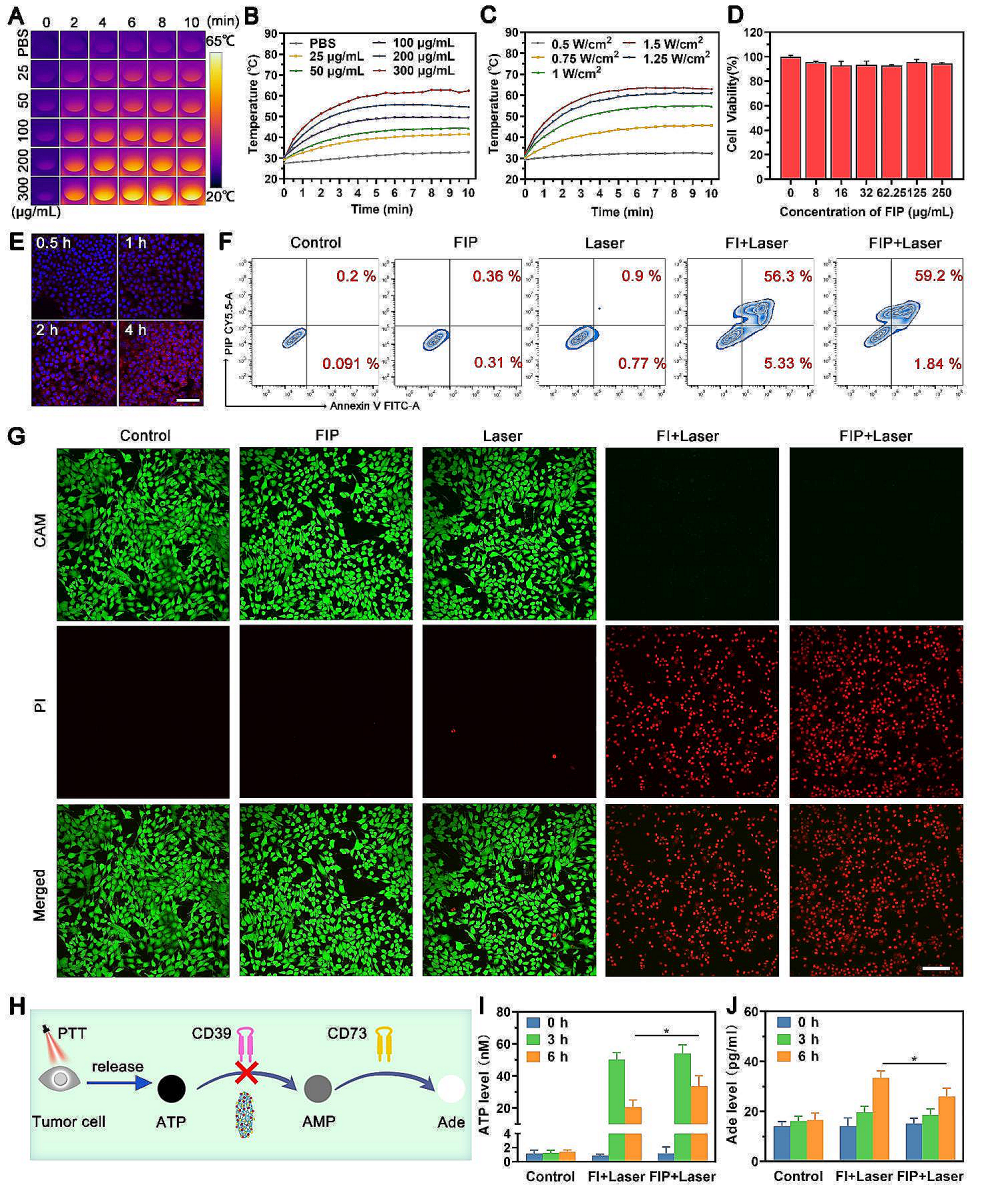
In vitro photothermal property and changes in ATP/Ade concentration. (**A**) Infrared thermal images and (**B**) temperature change curves of FIP at different concentrations. (**C**) Temperature change curves of FIP at different powers. (**D**) Relative viability of 4T1 cells after incubation with FIP for 24 h. (**E**) Intracellular uptake of FIP exanimated by CLSM after various incubation time. Scale bar: 100 μm. (**F**) Flow cytometry apoptosis assay stained after varying treatments. (**G**) 4T1 cells were co-stained with PI and Calcein-AM, following different treatments observed by CLSM. Scale bar: 100 μm. (**H**) Schematic illustration of Ade metabolism treated by FIP. (**I**-**J**) ATP and Ade levels in 4T1-cell culture medium after different treatments

The in vitro photothermal performance of the FIP was assessed by measuring the temperature changes inside a PBS solution. Following exposure to the 808 nm laser (1.5 W/cm^2^, 10 min), the temperature of the FIP solutions showed a fast rise, reaching around 60℃ (Fig. 3A and B). The temperature variations were increasing in correlation with the elevated concentrations of FIP. Furthermore, the photothermal effect may readily be modified by adjusting the laser intensity (Fig. 3C and Additional file 1: Figure S9). Besides, the FIP and FI showed similar photothermal performance (Additional file 1: Figure S10). Next, we investigated the in vitro cytotoxicity of FIP toward 4T1 cells by a standard cell counting kit-8 (CCK-8) assay. The result showed that FIP exhibited no evident cytotoxicity when tested on 4T1 cells, even at a high concentration of 250 µg/mL (Fig. 3D). Subsequently, we investigated the intracellular uptake performance of FIP. As exhibited in Fig. 3E, with increasing coincubation time, the red fluorescence (FIP doped with Ce6) gradually increased within the 4T1 cells. Flow cytometry results were consistent with the CLSM results (Additional file 1: Figure S11). These observations suggested that FIP could be readily internalized by 4T1 cells in a time-dependent pattern.

The photothermal effect of FIP on cancer cells was assessed by flow cytometry. As expected, both FI and FIP displayed obvious cytotoxic toward 4T1 cells when subjected to 808 nm laser irradiation (1.5 W/cm^− 2^, 10 min), suggesting that ICG is responsible for the photothermal killing effect rather than POM-1 (Fig. 3F and Additional file 1: Figure S12). Additionally, the photothermal effect of FI and FIP on 4T1 cells was intuitively evaluated with the calcein-AM and propidium (PI) co-staining method. The results showed that the control, laser, and FIP group showed minimal red fluorescence, suggesting that neither the NIR laser nor the FIP alone showed a noticeable lethal effect on the 4T1 cells (Fig. 3G). The FI + Laser and FIP + Laser groups exhibited a more pronounced red fluorescence than others. The above results suggested that the FIP-mediated PTT could kill cancer cells effectively.

Encouraged by the excellent PTT-killing effect of FIP, we further investigated ICD caused by FIP-mediated PTT and dual-directional immunometabolic regulating ability (Fig. 3H). As exhibited in Fig. 3I, the ATP level was significantly elevated at similar levels in the FI + Laser group and FIP + Laser group at 3 h after laser irradiation. At 6 h after laser irradiation, a significant decrease in ATP level was observed in the FI + Laser group and FIP + Laser group, and the ATP level of the FIP + Laser group was significantly higher than that of the FI + Laser group. The results could be explained by PTT-induced ICD releasing ATP and reduced ATP breakdown in the presence of POM-1. Besides, the Ade level of the FI + Laser group was significantly higher than the control group and FIP + Laser group at 6 h after laser irradiation (Fig. 3J), indicating the PTT could enhance the Ade generation and POM-1 could inhibit the Ade generation. These results suggested that the introduction of POM-1 effectively hindered the breakdown of ATP and the generation of Ade, therefore reversing the state of immunosuppression.

### PA and photothermal imaging of FIP

**Fig. 4:**
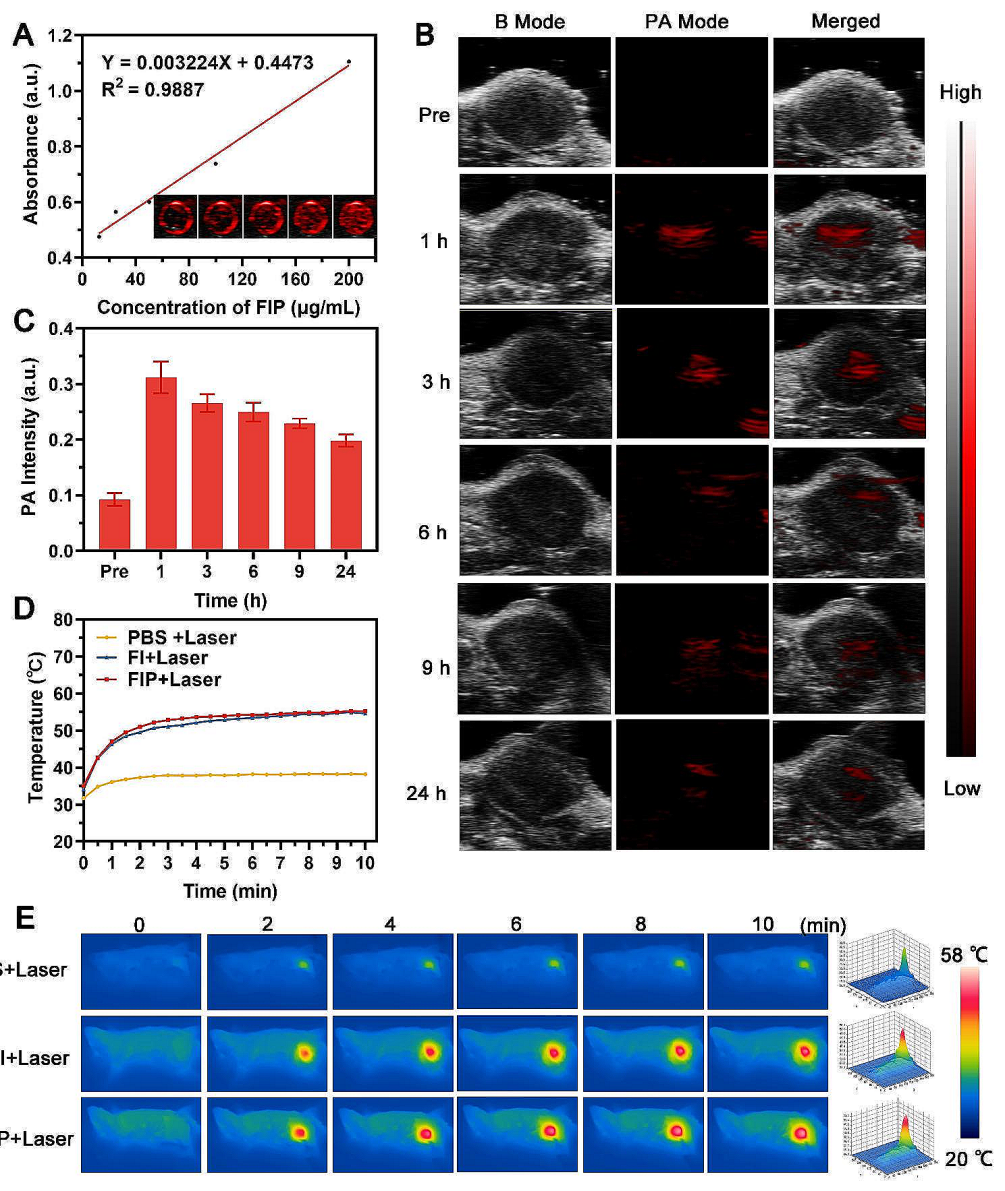
PA and photothermal imaging of FIP. (**A**) PA image of FIP at increased concentrations and the corresponding PA signal intensities. (**B**) PA images of tumor tissues after intravenous administration of FIP at different time points and (**C**) the corresponding signal intensities. (**D**-**E**) Temperature-elevation curves and corresponding infrared thermal images of tumors after various treatments

The optimal nanomedicine is projected to provide accurate imaging guidance and effective tumor therapy while also enabling the evaluation of therapeutic outcomes [38]. Due to nanomedicine’s strong absorption of NIR light is strongly associated with the photoacoustic (PA) effect, which is helpful for imaging purposes [39, 40]. Therefore, FIP was investigated as a contrast agent to improve the PA signal. The in vitro PA signal intensities of FIP aqueous dispersion at elevated concentrations were evaluated at a wavelength of 810 nm, demonstrating a clear correlation between the signal intensities and the concentration of the FIP (Fig. 4A). The tumor’s PA signal intensity increased following the delivery of FIP and peaked at 1 h after injection, indicating a substantial accumulation of FIP (Fig. 4B and C). The in vivo PA imaging result emphasized the advantages of FIP in accurately tracking the optimal time points of tumor accumulation.

Based on superb in vitro photothermal performance and optimal time points of tumor accumulation provided by PA imaging, we further explored the in vivo photothermal performance of FIP through photothermal imaging. As shown in Fig. 4D, the temperature at the tumor site rapidly increased to approximately 55℃ within 10 min in both the FI + Laser group and the FIP + Laser group. Conversely, the temperature in the laser alone group had a much lower peak temperature, as shown by an infrared camera (Fig. 4E). These results demonstrated that FI and FIP exhibited excellent photothermal performance in vivo.

### Evaluation of the effectiveness of immunometabolic regulator FIP-assisted photothermal immunotherapy in vivo

**Fig. 5:**
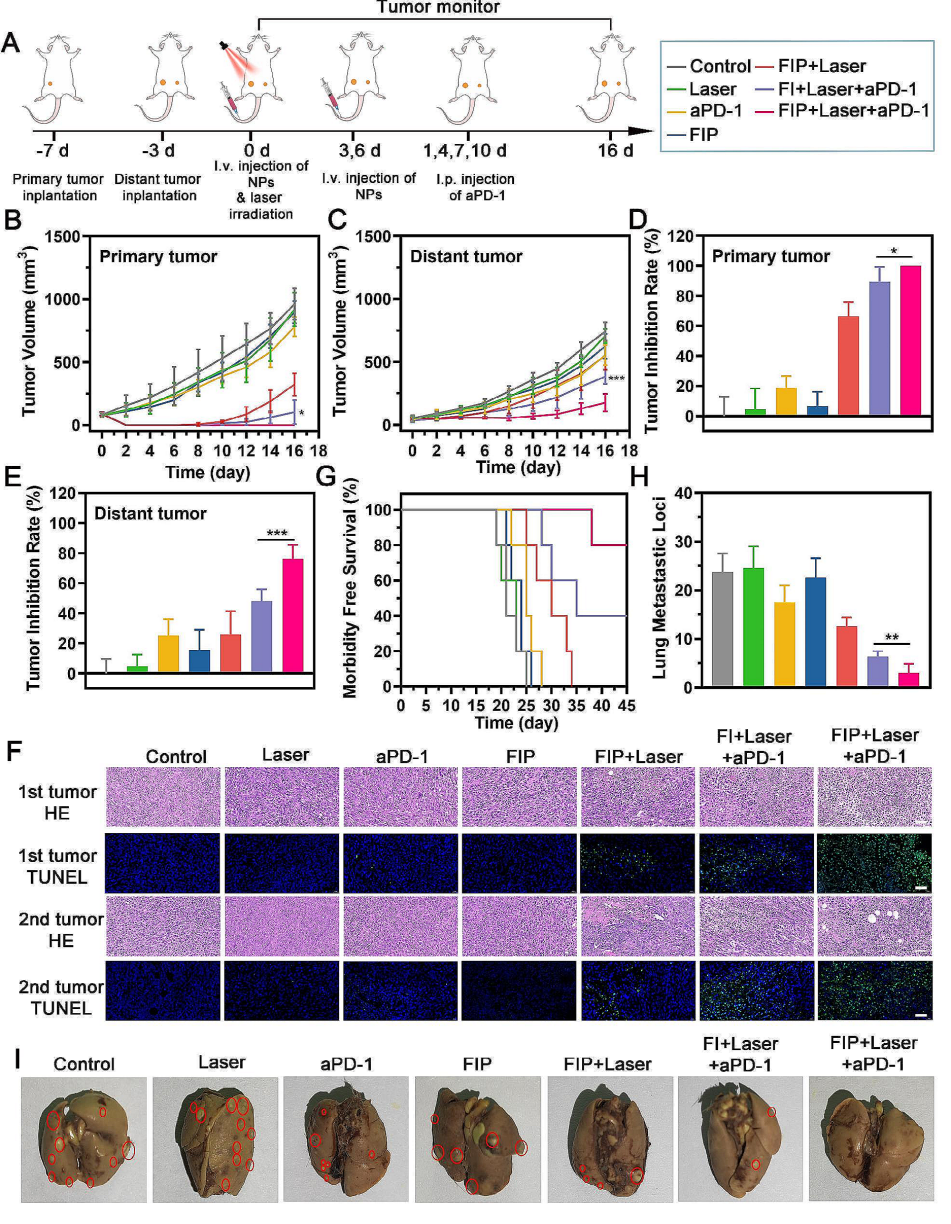
Therapeutic efficacy of immunometabolic regulator FIP-assisted photothermal immunotherapy in vivo. (**A**) Schematic illustration of the experiment design for evaluating the therapeutic effect in vivo. (**B**-**C**) Time-dependent tumor growth curves of primary tumors and distant tumors. (**D**-**E**) Tumor inhibition rates of primary tumors and distant tumors. (**F**) Pathology result of the primary and distant tumors stained by H&E and TUNEL. Scale bar: 50 μm. (**G**) Morbidity-free survival of mice following various treatments. (**H**-I) Gross appearance and the number of pulmonary metastasis after different treatments

We examined the potential of immunometabolic regulator FIP-assisted photothermal immunotherapy with a 4T1 tumor model, as shown in a schematic illustration (Fig. 5A). Our investigation unveiled that both the primary and distant tumors of the FIP + Laser + aPD-1 group showed the most substantial inhibition effect compared to other groups (Fig. 5B and E and Additional file 1: Figure S13). This confirmed the remarkable efficiency of immunometabolic regulator-enhanced photothermal immunotherapy in inhibiting tumor growth. Of note, the primary tumors of FIP + Laser + aPD-1 group were inhibited entirely for 16 days, while the primary tumors of FI + Laser + aPD-1 group only exhibited a short-term inhibition (8-day monitoring period) and an apparent tumor recurrence with the prolonged monitoring period to 16 days. Similarly, the distant tumors of the FIP + Laser + aPD-1 group showed a more substantial inhibition effect than the FI + Laser + aPD-1 group. These results strengthened the idea that FIP could enhance photothermal immunotherapy by limiting ATP degradation and preventing the formation of Ade. No noticeable body weight change was recorded throughout the observation period among these groups, indicating the apparent biosafety of the therapeutic process (Additional file 1: Figure S14). The tumor sections were subjected to Hematoxylin-eosin (H&E) and TdT-mediated dUTP nick-end labeling (TUNEL) staining. This analysis indicated that the FIP + Laser + aPD-1 group exhibited the highest levels of apoptosis and necrosis in the tumors (Fig. 5F). According to the survival curve, 80% of the mice receiving FIP-reinforced photothermal + aPD-1 treatment were still alive on day 45, almost double the survival rate of the other groups (Fig. 5G). The photograph of lung metastasis revealed that the FIP + Laser + aPD-1 group had the lowest incidence of lung metastasis (Fig. 5H and I). Therefore, we were convinced that the immunometabolic regulator FIP could significantly assist in potent photothermal immunotherapy. Moreover, applying the Ade inhibitory method significantly improved the efficacy of photothermal therapy in combination with immune checkpoint blockade (ICB). This methodology was anticipated to be more effective than traditional photothermal immunotherapy.

### Study into the immune mechanisms

Considering that tumor metastasis is the primary cause of death after PTT for cancer, an ideal PTT should not only locally impede the residual tumor but also detect, inhibit, and eradicate the metastases [41, 42]. Immunotherapy utilizing ICB has shown a notable surge in usage in recent years [43–45]. Specifically, integrating PD-1/PD-L1 checkpoint inhibition with other treatment protocols, such as PTT, has attracted significant interest in scientific investigation [46, 47]. Given the significant association between Ade and clinical ICB resistance, along with the observed excessive production of Ade in tissues after incomplete PTT, we were interested in examining whether the combination of FIP and laser treatment could enhance sensitivity to PD-1 blocking.

**Fig. 6:**
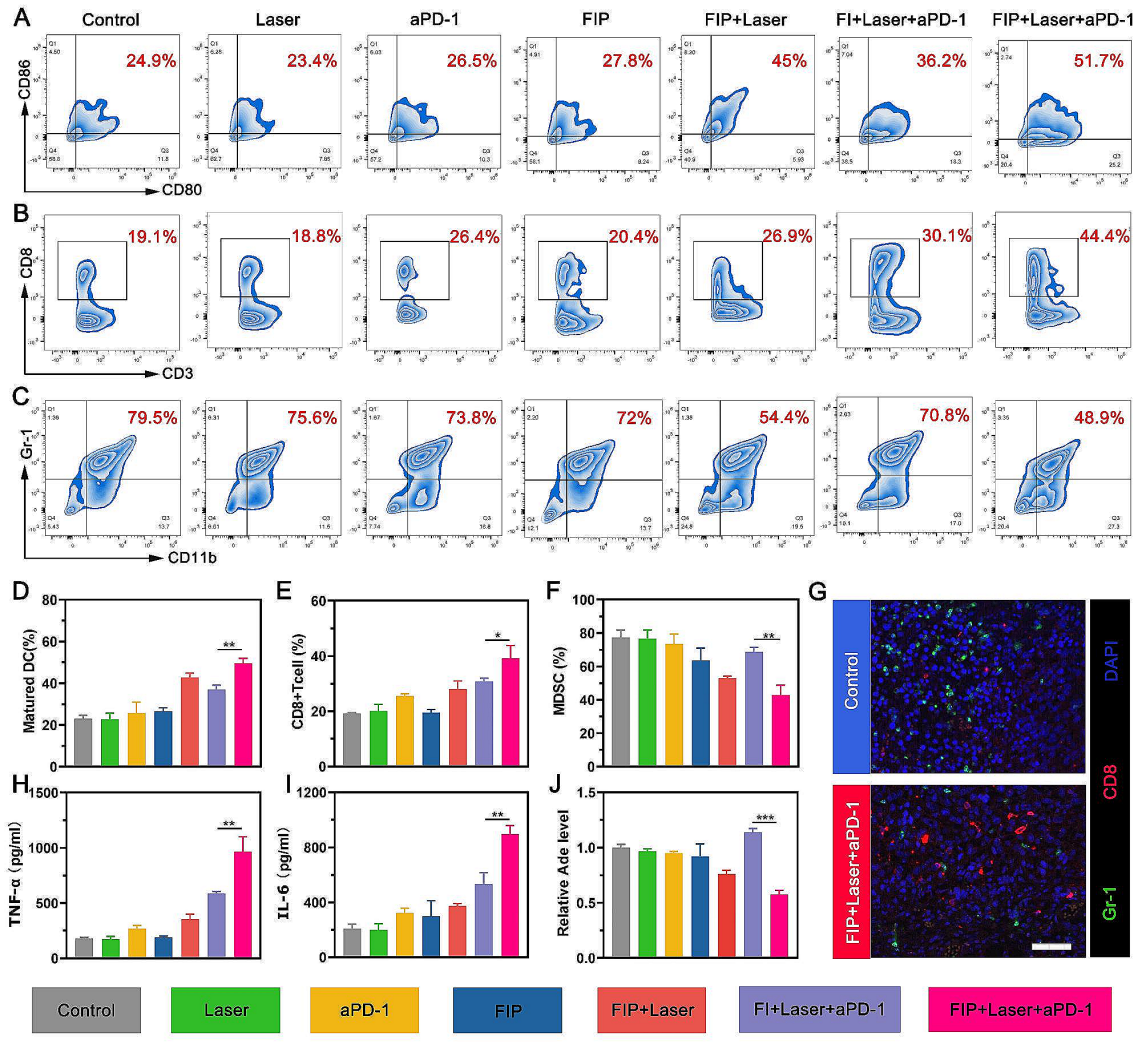
Study into the immune mechanisms. (**A**) Representative flow cytometric analysis of matured DCs (CD80^+^CD86^+^) in tumor-draining lymph nodes and (**D**) relative quantitative data. (**B**) Representative flow cytometric analysis of CD8 + T cells (CD3^+^CD8^+^) and (**E**) relative quantitative data. (**C**) Representative flow cytometric analysis of MDSC cells (CD45^+^CD11b^+^Gr-1^+^) and (**F**) relative quantitative data. (**G**) Representative polychromatic immunofluorescent staining images of tumors showing DAPI (blue), CD8+ (red), and Gr-1+ (green) cell infiltration for Control and FIP + Laser + aPD-1 groups. Scale bar: 50 μm. (**H**-**J**) After different treatments, TNF-α, IL-6, and Ade levels in the primary tumors

Our study found that the proportion of mature DCs in the FIP + Laser + aPD-1 group was 51.7%, which was significantly higher than that of other groups (Fig. 6A). This discovery provided evidence that FIP-assisted photothermal immunotherapy could effectively induce ICD and promote DC maturation for enhanced antigen presentation. CD8 + T cells, as cytotoxic T lymphocytes (CTLs), can eradicate cancerous cells directly and are pivotal in the immune response to tumors [48, 49]. Our results found that the FIP + Laser + aPD-1 group had a proportion of CD8 + T cells of 44.4%, which was much higher than the aPD-1group (26.4%), FIP + Laser group (26.9%) and FI + Laser + aPD-1 group (30.1%) (Fig. 6B). This discovery suggested that FIP-assisted photothermal immunotherapy could boost anti-tumor immune response and highlighted the need to reduce the Ade level in photothermal immunotherapy. MDSCs can protect tumor cells from immune system assaults and impede the immune response against tumors [50, 51]. Our results showed that the FIP + Laser + aPD-1 group had a much-reduced proportion of MDSCs compared to other groups (Fig. 6C), suggesting that the tumor’s immunosuppressive environment was significantly relieved. To summarize, after introducing POM-1, the group treated with FIP + Laser + aPD-1 showed higher levels of mature DCs and CD8 + T cells and lower levels of MDSCs (Fig. 6D and F). The multiple immunofluorescence staining demonstrated consistent outcomes (Fig. 6G). TNF-α and IL-6, endogenously generated cytokines, serve as crucial biomarkers that signify the magnitude of the immune response. Our study results showed that the concentrations of TNF-α and IL-6 were notably increased in the FIP + Laser + aPD-1 group compared to other groups (Fig. 6H and I), indicating an intense immune response to the tumor generated by FIP + Laser + aPD-1. It was worth mentioning that the Ade level of FI + Laser + aPD-1 was significantly higher than other groups (Fig. 6J). The concurrent application of FIP, laser, and aPD-1 treatment resulted in a substantial reduction in Ade levels compared to that of the FI + Laser + aPD-1 group. These findings indicated that FIP as immunometabolic regulator-assisted photothermal immunotherapy could successfully inhibit the generation of Ade after PTT and promote a robust immune response. As a result, this treatment approach alleviated immunosuppression and enhanced the tumor’s responsiveness to aPD-1.

### Immune memory effect

**Fig. 7:**
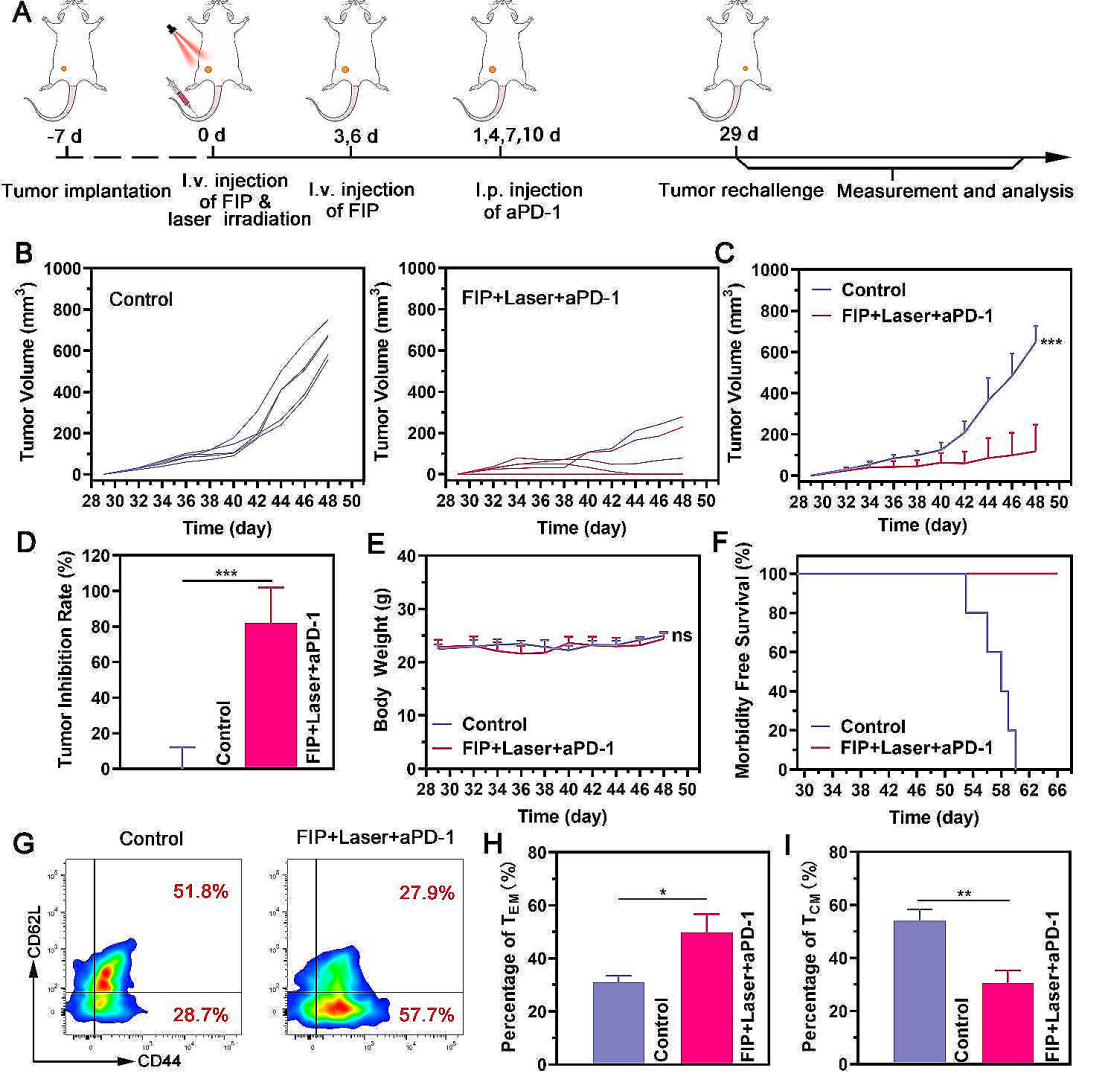
Immune memory effect. (**A**) Schematic illustration of the experiment design for the rechallenged tumor model. (**B**) Individual growth curves of rechallenge tumor in each group. (**C**) Average growth curves of rechallenge tumor in each group. (**D**) Tumor inhibition rate after different treatments. (**E**) Body weight data in mice after different treatments. (**F**) Morbidity-free survival of mice after various treatments. (**G**-**I**) Representative flow cytometric analysis of T_EM_ and T_CM_ (gated on CD3^+^CD8^+^ T cells) in the spleens the day before tumor rechallenging and the corresponding quantitative data

The immune memory effect is a crucial characteristic of adaptive immunity, providing a durable defense against future pathogen invasions [52]. This response is critical in preventing tumor recurrence. In light of this, we investigated the immune memory effect in a tumor recurrence model (Fig. 7A). As shown in Fig. 7B and D, compared to the control group, the rechallenged tumors of the FIP + Laser + aPD-1 group were remarkably suppressed with two complete tumor eradication among the five mice, indicating the impressive immune memory effect of FIP-assisted photothermal immunotherapy. No substantial body weight reduction was reported in mice following the therapy (Fig. 7E). Furthermore, all FIP + Laser + aPD-1 group mice survived for 66 days (Fig. 7F), demonstrating that the proposed photothermal immunotherapy provided long-lasting protection against tumor recurrence.

It has been reported that antigen-specific memory T cells can be categorized into two subsets: central memory (T_CM_) and effector memory T cells (T_EM_). T_CM_ (CD3 + CD8 + CD62L + CD44+) can protect the body only after a series of antigen-stimulated clonal expansion, differentiation, and migration. On the other hand, T_EM_ (CD3 + CD8 + CD62L-CD44+) can rapidly respond and offer immediate protection when exposed to the same tumor antigen [53]. Therefore, the shift from T_CM_ to T_EM_ may indicate a more robust anti-tumor immune response [54, 55]. In our study, it was found that the mice treated with FIP + Laser + aPD-1 showed a significant increase in the percentage of T_EM_ cells and a substantial decrease in T_CM_ cells compared to the mice who underwent surgery, indicating a robust immune memory effect for tumor prevention (Fig. 7G and I). Therefore, these studies provided crucial evidence that a robust and durable immune response against tumors had been formed by FIP-assisted photothermal immunotherapy.

### Biosafety assay of FIP

Afterward, we assessed the biosafety of FIP as a multipurpose nanocarrier in both experiments and live mice. The occurrence of hemolysis in blood cells remained below 5% even at a concentration of 200 µg/mL of FIP (Additional file 1: Figure S15). In addition, the serum biochemical tests did not reveal any abnormal alterations, and the hematoxylin and eosin (H&E) staining of the major organs showed no noticeable pathological abnormalities compared to the normal mice (Additional file 1: Figure S16-17). This suggested that the FIP had great histocompatibility. Our findings demonstrated that FIP was highly biocompatible as a promising therapeutic agent in vivo.

## Conclusions

In conclusion, our research presents a groundbreaking approach to overcoming the challenges associated with incomplete PTT for cancer treatment. We have identified the critical immunosuppressive mechanisms within the TME that facilitate tumor recurrence and metastasis post-PTT. Our findings demonstrate the critical role of the CD39-mediated ATP-Ade pathway in promoting an immunosuppressive environment that diminishes the effectiveness of ICB. By integrating photothermal agents with immunometabolic regulators, we offer a novel solution that enhances PTT’s efficacy and transforms the immunosuppressive TME into an immunogenic one. Significantly, it can inhibit the ATP-adenosine pathway by dual-directional immunometabolic regulation, resulting in increased ATP levels and decreased adenosine synthesis. This dual-directional strategy significantly improves PTT-immunotherapy outcomes by leveraging the synergistic effects of enhanced PTT and immunometabolic regulation. With the aid of aPD-1, the FIP effectively suppresses tumor growth and prevents recurrence. This approach not only addresses the limitations of current PTT but also offers a new avenue for the comprehensive treatment of cancer. Our work highlights the importance of dual-directional immunometabolic regulation with nanomedicine as a pivotal strategy for enhancing the effectiveness of cancer therapies.

### Methods and materials

#### Materials

FeCl3·6H2O, or Fe (III) chloride hexahydrate, was purchased from Thermo Fisher Scientific (USA). Adamas-Beta produced diammonium pyridine (DAP). The supplier of ICG was Macklin (China). POM-1 was purchased from MCE (United States). aPD-1 (clone: RMP1-14, catalog number: BE0146) was acquired from BioxCell. Boster Biological Technologies (China) was the source of DAPI. We purchased Calcein-AM and PI from Dojindo (Japan). Fetal bovine serum (2%) was purchased from Pan (Germany), and collagenase IV (0.02%), and DNase I (0.002%) were obtained from Solarbio (China). FITC-CD80 (catalog: 104,705), APC-CD11c (catalog: 117,309), live-dead BV421 (catalog: 423,114), PE-CD86 (catalog: 105,008), FITC-CD3 (catalog: 100,203), APC-Cy7-CD45 (catalog: 103,116), PE-Cy7-CD11b (catalog: 101,215), APC-CD8 (catalog: 100,711), and PE-Gr-1 (catalog: 108,407) were obtained from Biolegend. Percp-Cy5.5-CD44 (catalog: 45-0441-80) and PE-CD62L (catalog: 12-0621-81) were purchased from eBioscience.

### Cell and animal

The 4T1 murine breast cancer cells were obtained from Chongqing Medical University. The 4T1 cells were cultured in RPMI 1640 complete media containing 10% FBS and 1% penicillin-streptomycin. The cells were maintained in a humidified environment with 5% CO2 at a temperature of 37 °C.

The Animal Center of Chongqing Medical University supplied female BALB/c mice aged 6–8 weeks. All the animal experiments were conducted following the authorized protocols of the Animal Ethics Committee of Chongqing Medical University.

### Synthesis of FI

First, Fe-PDAP was produced using an oxidative polymerization process induced by Fe (III). In summary, a solution of FeCl3·6H2O (10 g) was prepared by dissolving it in 200 mL of distilled water and subjecting it to sonication for 10 min. A quantity of DAP weighing 1.0 g was introduced into the mixture and agitated for 24 h (37 °C) to initiate polymerization. The resulting mixture was subsequently filtered using a dialysis bag with a molecular weight cutoff of 12 kDa by immersing it in distilled water overnight. Subsequently, Fe-PDAP was acquired and subsequently kept at a temperature of 4 °C for further use. Secondly, ICG (10 mg) was evenly spread in Fe-PDAP (50 mg) which was dissolved in deionized water. After that, the mixture was shaken with a magnet for a whole night at the ambient temperature shielded from light. Afterward, the production was collected after repeated centrifugations (12,000 g, 10 min).

### Characterization of FI

The TEM pictures were acquired using an FEI Tecnai G2 F20 microscope (USA). The SEM pictures were acquired using ZEISS GeminiSEM 300 (Germany). The Zetasizer (Malvern Instruments, UK) was used to measure the particle size and zeta potential. The spectrophotometer used to get the UV-Vis absorption spectra was the Multiskan Go (Thermo Fisher Scientific).

### Increased generation of Ade after incomplete PTT deteriorates immunosuppressive microenvironment

Cell suspensions of 4T1 cells were injected into the right mammary fat pad of each animal to create an orthotopic breast cancer model with the primary tumor. Once the tumor reached 50–100 mm^3^, the mice were randomly divided into three groups. On day 0, the animals were given intravenous injections of NPs at 3 mg/mL. The groups were exposed to 1.5 W/cm^2^ laser irradiation for 5 min. The tumor size was assessed using a caliper every other day, and the volume was determined using the formula (width2 × length)/2.

To explore processes behind the immune suppression caused by incomplete PTT, 4T1 tumors were obtained at 3 days after laser irradiation, and Ade level was analyzed with ELISA kits from Fankew company. To further explore the underlying mechanism of Ade production, we performed ATP and Ade analysis in vitro. The cells were cultivated in 24-well plates and left to incubate with different NPs for 4 h. Next, the cells underwent laser irradiation. After the indicated incubation time (3 h and 6 h), the liquid portion of the cell culture was collected and analyzed using an Enhanced ATP Assay Kit (Beyotime, China) and an Ade ELISA kit following the instructions provided by the manufacturers.

To analyze the infiltration of immune cells in residual 4T1 tumors, the mice were euthanized and the tumors were obtained. The tumors were immediately split into smaller pieces and treated with a solution containing fetal bovine serum (2%), collagenase IV (0.02%), and DNase I (0.002%). The mixture was physically disrupted with a syringe’s rubber end to obtain a single-cell suspension and passed through a 40 μm nylon cell strainer. Following the removal of red blood cells with related lysis solution and washing with PBS, the individual cells were treated with a fluorescently tagged antibody as directed by the manufacturer. To analyze CD8 + T cells, they were stained with live-dead BV421, APC-Cy7-CD45, FITC-CD3, and APC-CD8. The cells were stained with APC-Cy7-CD45, PE-Cy7-CD11b, and PE-Gr-1 to analyze MDSC.

### Synthesis of FIP

Concisely, a mixture of ICG (10 mg) and POM-1 (30 mg) was evenly spread in Fe-PDAP (50 mg) and dissolved in deionized water. After that, the mixture was shaken with a magnet for a whole night at the ambient temperature shielded from light. Afterward, the production was collected after repeated centrifugations (12,000 g, 10 min).

### Characterization of FIP

The FTIR results were obtained by Nicolet 670 (USA). XRD pictures were acquired using a BrukerAXS D8 (Germany). The XPS spectra were obtained using a Thermo Scientific K-Alpha instrument (USA). The same machines obtained all other characterization results, as shown above.

### Photothermal performance of FIP in vitro

The FIP solution was exposed to an 808 nm laser irradiation with the intensity of 1.5 W/cm^2^ at various concentrations. In addition, we subjected the FIP aqueous solution to an 808 nm laser with varying power intensities. The solution’s temperature and associated NIR images were recorded at 30-second intervals using a Fotric 226 infrared thermal imaging camera.

### In vitro cytotoxicity measurement of FIP

FIP’s cytotoxicity toward 4T1 cells was determined using the standard CCK-8 assay. Typically, 5 × 10^3^ cells were grown in 96-well plates per well for one night to allow adherence to the bottom of each well. Subsequently, varied doses of FIP were added and left to incubate for another 24 h. Then, the CCK-8 method was carried out, and the results were obtained using a multimode reader (Multiskan Go, Thermo Fisher Scientific).

### Intracellular uptake performance of FIP

Ce6 was employed to label FIP in our study. Briefly, a solution of Ce6 in methanol was mixed with a dispersion of Fe-PDAP in methanol and stirred overnight, and the free Ce6 was removed by ultrafiltration with a centrifugal filter of 100 kDa. Ce6-doped Fe-PDAP synthesized ce6-doped FIP to carry ICG and POM-1. The resultant mixture was then stirred overnight in the absence of light. Ce6-doped FIP products were produced by centrifugation at 12,000 g for 10 min. Before receiving FIP NP treatment, 4T1 cells were grown on culture plates reserved for CLSM for one additional night. Before being analyzed by CLSM (λex/ λem = 630 nm /670 nm), the cells were fixed for 15 min with 4% paraformaldehyde, stained with DAPI, and then cleaned with PBS.

### In vitro photothermal effect of FIP against 4T1 cells

The cell apoptosis investigation involved culturing 4T1 cells in a six-well plate for 24 h and subjecting them to several treatments: control, FIP, Laser, FI + Laser, and FIP + Laser. Following washing, the cells underwent trypsinization, centrifugation, and dispersion in PBS. Subsequently, they were treated with annexin V-FITC/PI for 15 min before conducting flow cytometry analysis.

The in vitro PTT effect was examined by CLSM, where calcein-AM and PI stained the live cells (green fluorescence) and dead cells (red fluorescence), respectively. Briefly, 4T1 cells were grown on CLSM-exclusive plates at a density of 1 × 10^5^ cells per dish overnight and treated to several treatments. The cells were then irradiated with a 1.5 W/cm^2^ laser for 10 min. The cells were then stained with calcein-AM and PI for 15 min before being examined using CLSM.

### In vitro changes in ATP/adenosine concentration

The cells were cultivated in 24-well plates and incubated with different NPs for 4 h. Next, the cells underwent laser irradiation (1.5 W/cm^2^, 10 min). After indicated incubation time (3 h and 6 h), the liquid portion of the cell culture was collected and analyzed using an Enhanced ATP Assay Kit (Beyotime, China) and an Adenosine ELISA kit (Fankew, China) following the instructions provided by the manufacturers.

### PA imaging

We performed PA imaging in vitro using increasing doses of FIP. To perform in vivo PA imaging, BALB/c mice bearing 4T1 tumors were given FIP intravenously at 3 mg/mL. The PA signals in the tumor locations were captured at certain time intervals after the injection.

### Photothermal imaging in vivo

BALB/c mice bearing 4T1 tumors were randomly assigned to three groups and exposed to NIR laser radiation: the PBS + Laser group, the FI + Laser group, and the FIP + Laser group. The mice were methodically given various substances, followed by irradiation with an NIR laser (1.5 W/cm^2^, 10 min) at 1 h after the injection. The tumor temperature and IR thermal images were monitored using a Fotric 226 infrared thermal imaging camera.

### Evaluation of the effectiveness of immunometabolic regulator FIP-assisted photothermal immunotherapy in vivo and underlying immune mechanism study

To generate the main tumor in an orthotopic breast cancer model, the right mammary fat pad of each animal was injected with 1 × 10^6^ 4T1 cell suspensions (the primary tumor). Four days later, the mice’s left mammary fat pads were implanted with 5 × 10^5^ 4T1 cell suspensions (the distant tumor). When the primary tumor reached 50–100 mm^3^, the mice were separated independently into seven groups and injected with different NPs (3 mg/mL) on days 0, 3, and 6. The groups were exposed to 1.5 W/cm^2^ laser irradiation for 10 min each. The mice received laser irradiation 1 h after injection. Mice received 100 µg of PD-1 antibodies intraperitoneally on days 1, 4, 7, and 10, respectively. Every other day, a Vernier caliper was used to take the tumors’ measurements, and the method for calculating their volume was width squared multiplied by length divided by two. The mice’s body weights were measured on the same day. When the tumor grew to 1500 mm^3^, the mice were euthanized. Lung tissues from mice were submerged in Bouin’s solution to determine the number of lung tumor nodules.

To examine the immunological mechanism of treatment, the mice were euthanized on day eight, and the primary tumors and lymph nodes that drained the tumors were removed. The lymph nodes that drain the original tumor were immediately split into smaller sections and stained with APC-CD11c, PE-CD86, and FITC-CD80. The preparation and staining process of the tumors were the same process as above. The primary tumors were collected, homogenized, and centrifuged for the ELISA assay to extract the liquid part on the eighth day. TNF-α and IL-6 were measured using ELISA kits from Dakewe Biotech. Adenosine level was analyzed using ELISA kits from Fankew company.

### Immune memory effects

A mouse orthotopic tumor was created by injecting 4T1 cells into the right mammary fat pad of mice, which served as the primary tumor. Once the tumor reached 50–100 mm^3^, the mice were randomly divided into two groups. Then, we removed the primary tumor with FIP + Laser + aPD-1 or surgery (control group). 19 days after the primary tumors were removed, 5 × 10^5^ 4T1 cells were rechallenged into the mice’s left flanks of mice contralateral to the primary tumor. The diameters of rechallenged tumors were measured three days after their introduction and thereafter documented every other day.

To investigate memory T cells in the spleen, the spleen was obtained the day before the rechallenge and thoroughly crushed to create a solution of individual cells using a syringe’s rubber tip. The particular cell culture was labeled with FITC-CD3, APC-CD8, Percp-Cy5.5-CD44, and PE-CD62L. The cells were evaluated using a flow cytometer (BD FACS Canto Plus), and the data was analyzed with FlowJo software.

### Hemolysis analysis

Different concentrations of FIP were mixed with the diluted mice RBC suspension (5% hematocrit) for 2 h and then centrifuged. The supernatant was acquired to detect the absorbance and then calculate the hemolysis rate. PBS was set as the negative control, and deionized water was set as the positive control.

### In vivo biosafety

FIP (3 mg/mL) was administered intravenously to healthy female Kunming mice aged 6–8 weeks. The control groups comprised untreated healthy female Kunming mice. The mice were killed at 1, 7, 14, and 21 days after the administration of FIP. We obtained blood samples from the heart, liver, lung, spleen, and kidney and performed serum biochemistry analysis, routine blood testing, and histological examination.

### Statistical analysis

Data is provided as mean ± SD, with statistical analysis using student’s t-test and one-way ANOVA. *p < 0.05, **p < 0.01, ***p < 0.001.

### Electronic supplementary material

Below is the link to the electronic supplementary material.

Supplementary Material 1

## Data Availability

No datasets were generated or analysed during the current study.
