# Supplementary material for: Potentiating dual-directional immunometabolic regulation with nanomedicine to enhance anti-tumor immunotherapy following incomplete photothermal ablation
Source: J Nanobiotechnology. 2024 Jun 24;22:364. doi: 10.1186/s12951-024-02643-w (PMC11194966; doi:10.1186/s12951-024-02643-w)
Supplement: Supplementary file 1 — Supplementary Material 1 [file 12951_2024_2643_MOESM1_ESM.docx]

Supporting information

Potentiating Dual-Directional Immunometabolic Regulation with Nanomedicine to Enhance Anti-Tumor Immunotherapy Following Incomplete Photothermal Ablation

Qinqin Jiang ^1#^, Bin Qiao ^1#^ , Jun Zheng ^1^ , Weixiang Song ^2^, Nan Zhang ^3^ , Jie Xu ^2^, Jia Liu ^2^ , Yixin Zhong ^2^ , Qin Zhang ^4^, Weiwei Liu ^1^ , Lanlan You ^5^, Nianhong Wu ^1^, Yun Liu ^2^ , Pan Li ^1^ , Haitao Ran ^1^ , Zhigang Wang ^1^* and Dajing Guo ^2^*

^1^ Department of Ultrasound, Chongqing Key Laboratory of Ultrasound Molecular Imaging, the Second Affiliated Hospital of Chongqing Medical University, Chongqing 400010, P. R. China

^2^ Department of Radiology, the Second Affiliated Hospital of Chongqing Medical University, Chongqing 400010, P. R. China

^3^ Department of Medical Ultrasonics, the First Affiliated Hospital of Sun Yat-sen University, Guangzhou, 510080, P. R. China

^4^ Department of Radiology, Chongqing Hospital of Traditional Chinese Medicine, Chongqing 400021, P. R. China

^5^ Department of Ultrasound, Clinical Medical College and the First Affiliated Hospital of Chengdu Medical College, Chengdu, 610500, P. R. China

Qinqin Jiang and Bin Qiao are co-first authors who contributed equally to this work.

Corresponding authors: Dajing Guo, E-mail: [guodaj@hospital.cqmu.edu.cn](mailto:guodaj@hospital.cqmu.edu.cn)

Zhigang Wang, E-mail: wzg62942443@163.com


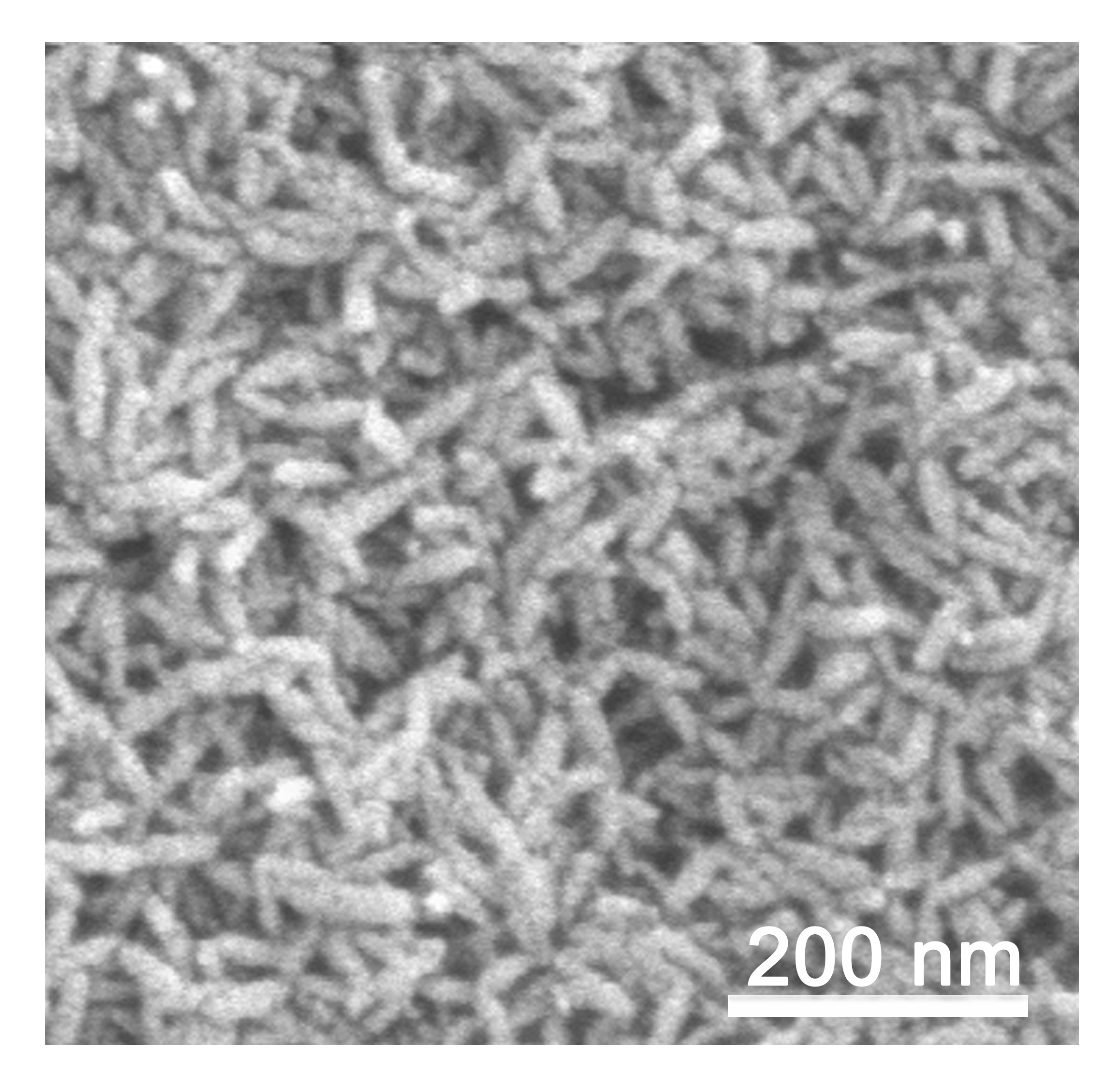


**Figure S1** SEM image of FI.


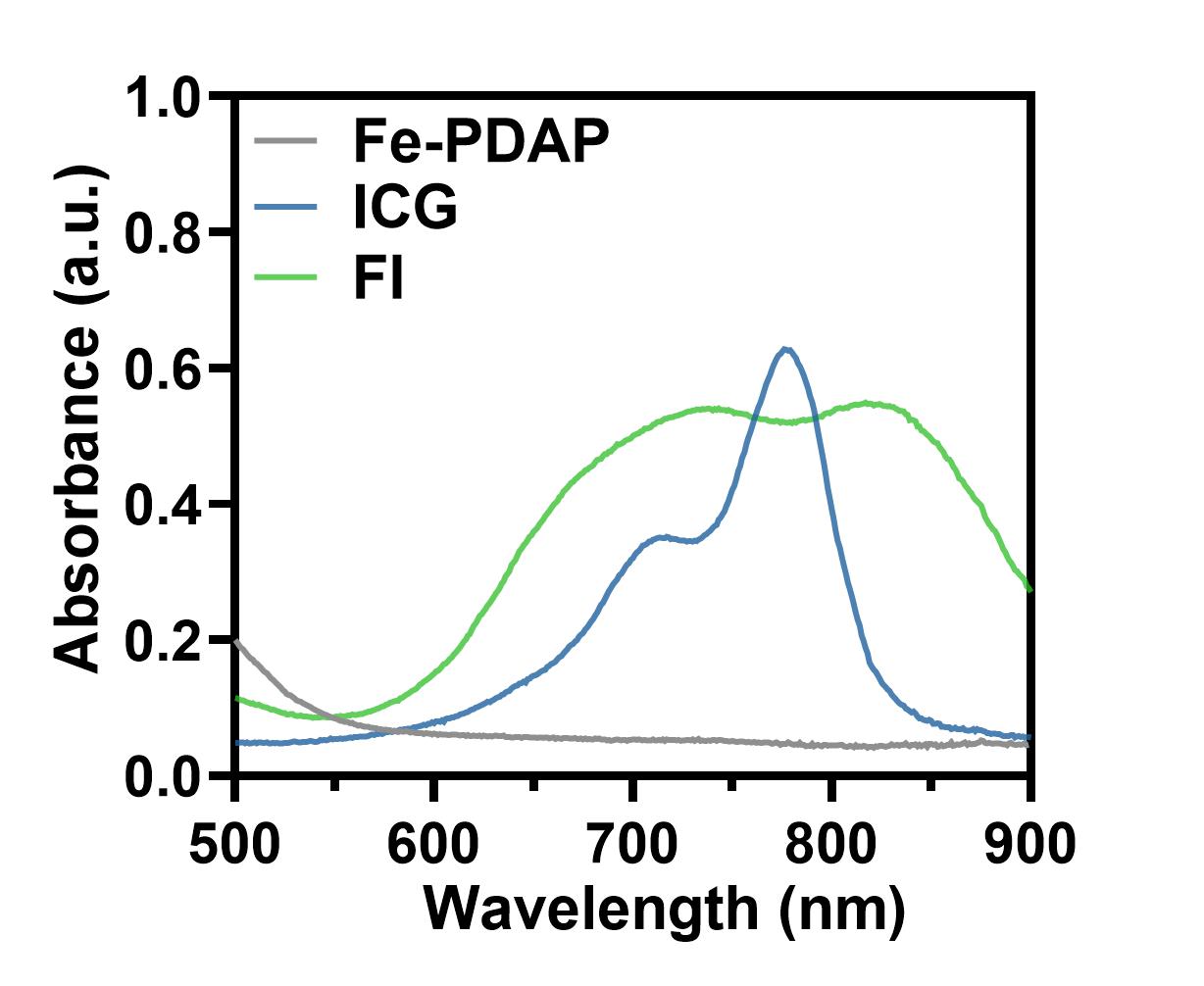


**Figure S2** UV/vis absorption spectra of Fe-PDAP, ICG and FI.

**
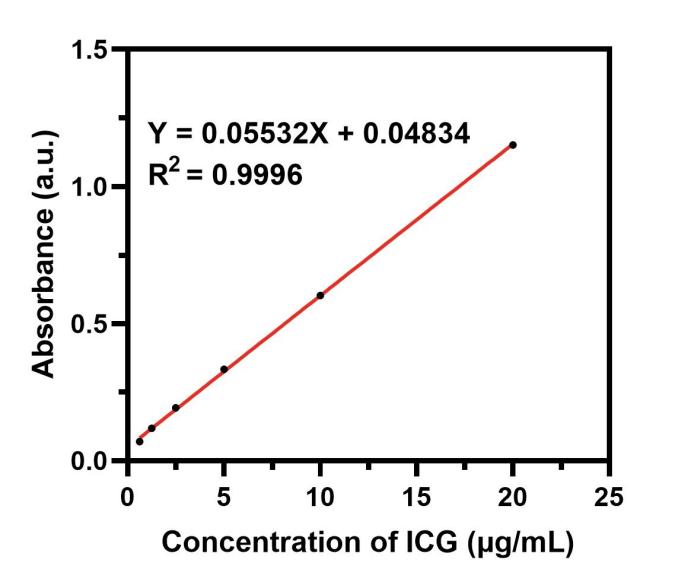
**

**Figure S3** The relative absorbance intensity of ICG in the UV-vis-NIR spectrum at a wavelength of 776nm.


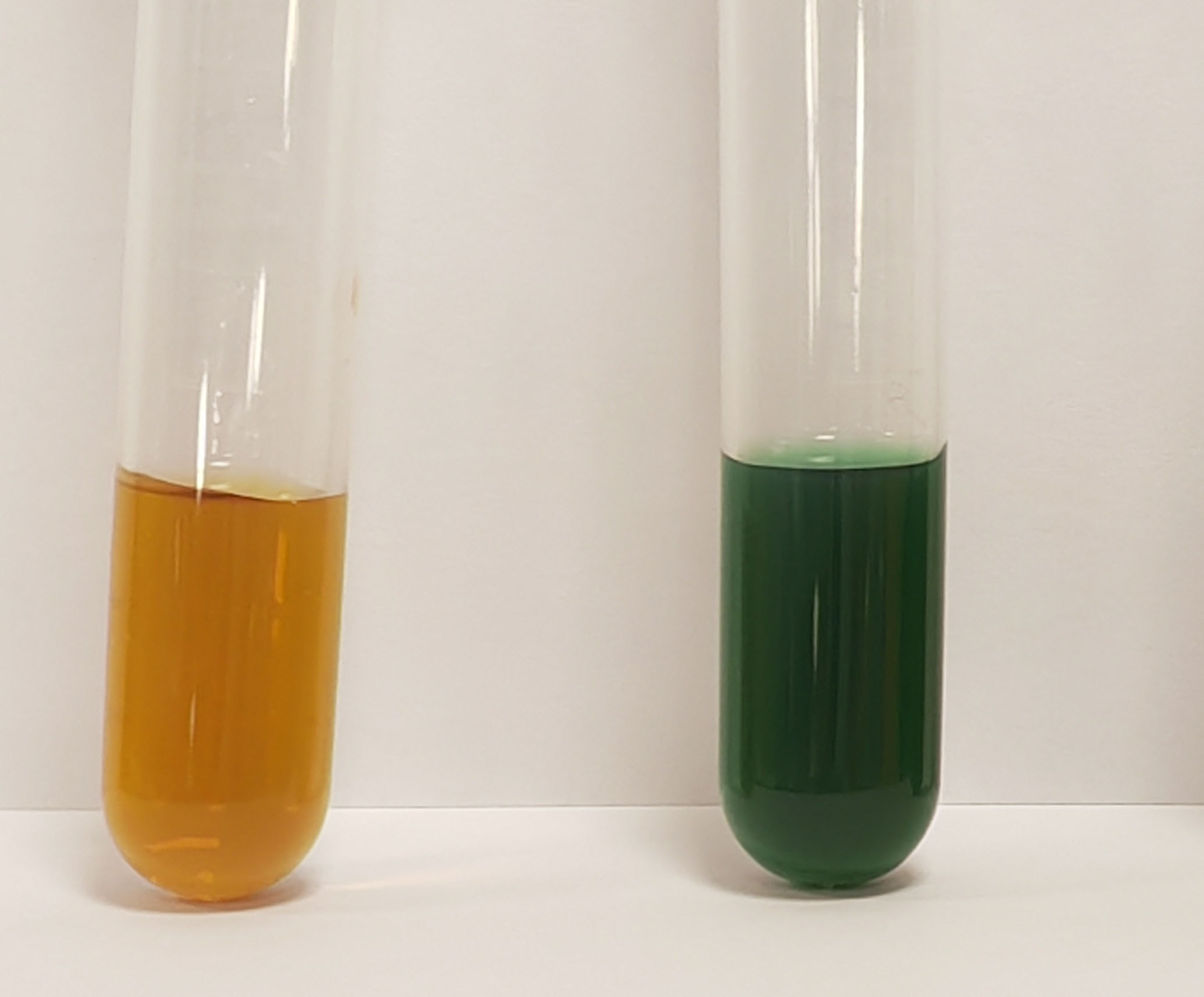


**Figure S4** Digital images of Fe-PDAP (left) and FI (right).


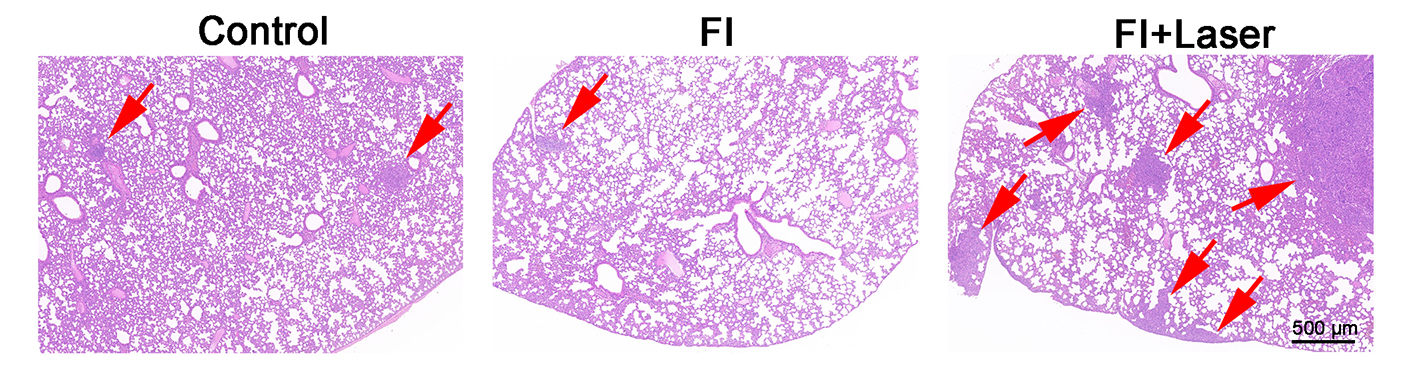


**Figure S5** Representative H&E staining images of lung tissues collected at the end of the study.


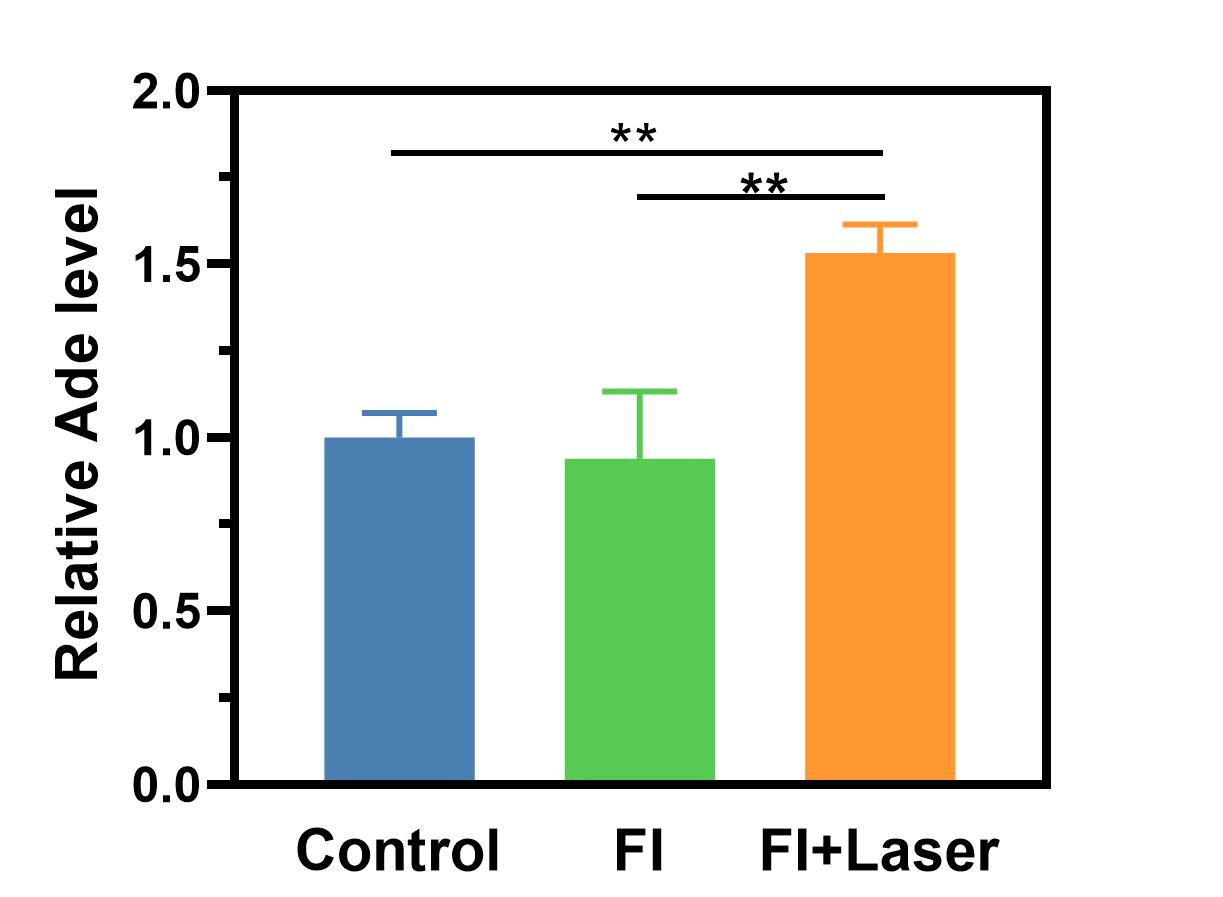


**Figure S6** Ade level in the tumor tissue after different treatments.

**
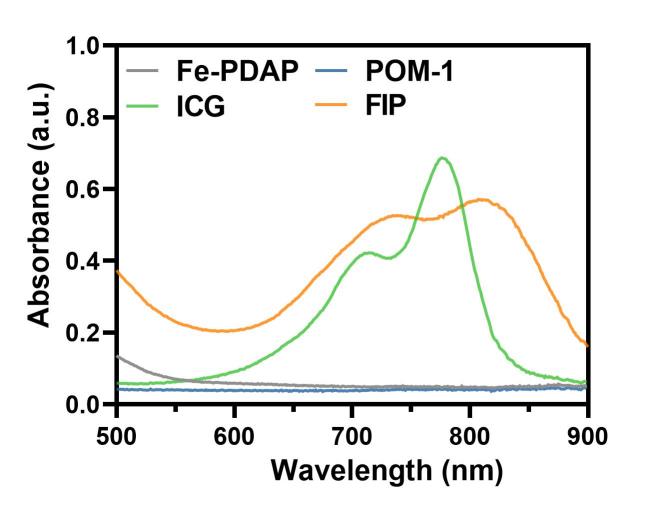
**

**Figure S7** UV/vis absorption spectra of Fe-PDAP, ICG, POM-1 and FIP.


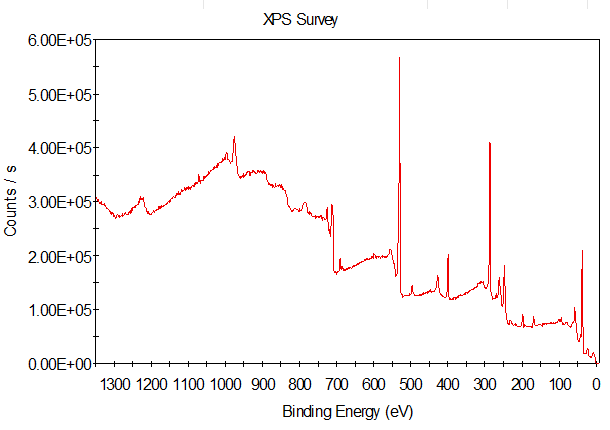


**Figure S8** Full survey XPS spectrum of FIP.

**
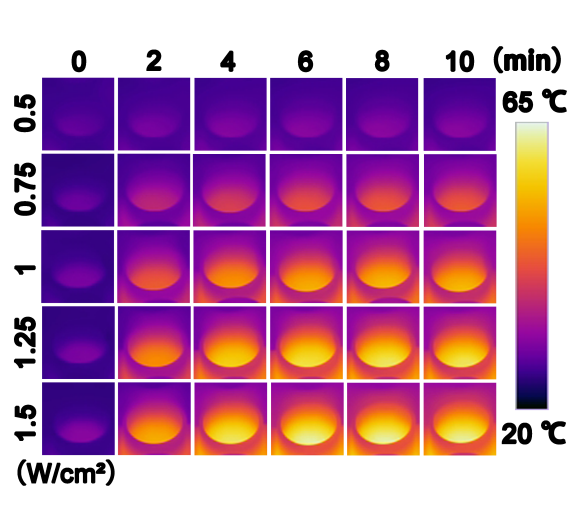
**

**Figure S9** Infrared thermal images in vitro of FIP at various powers.


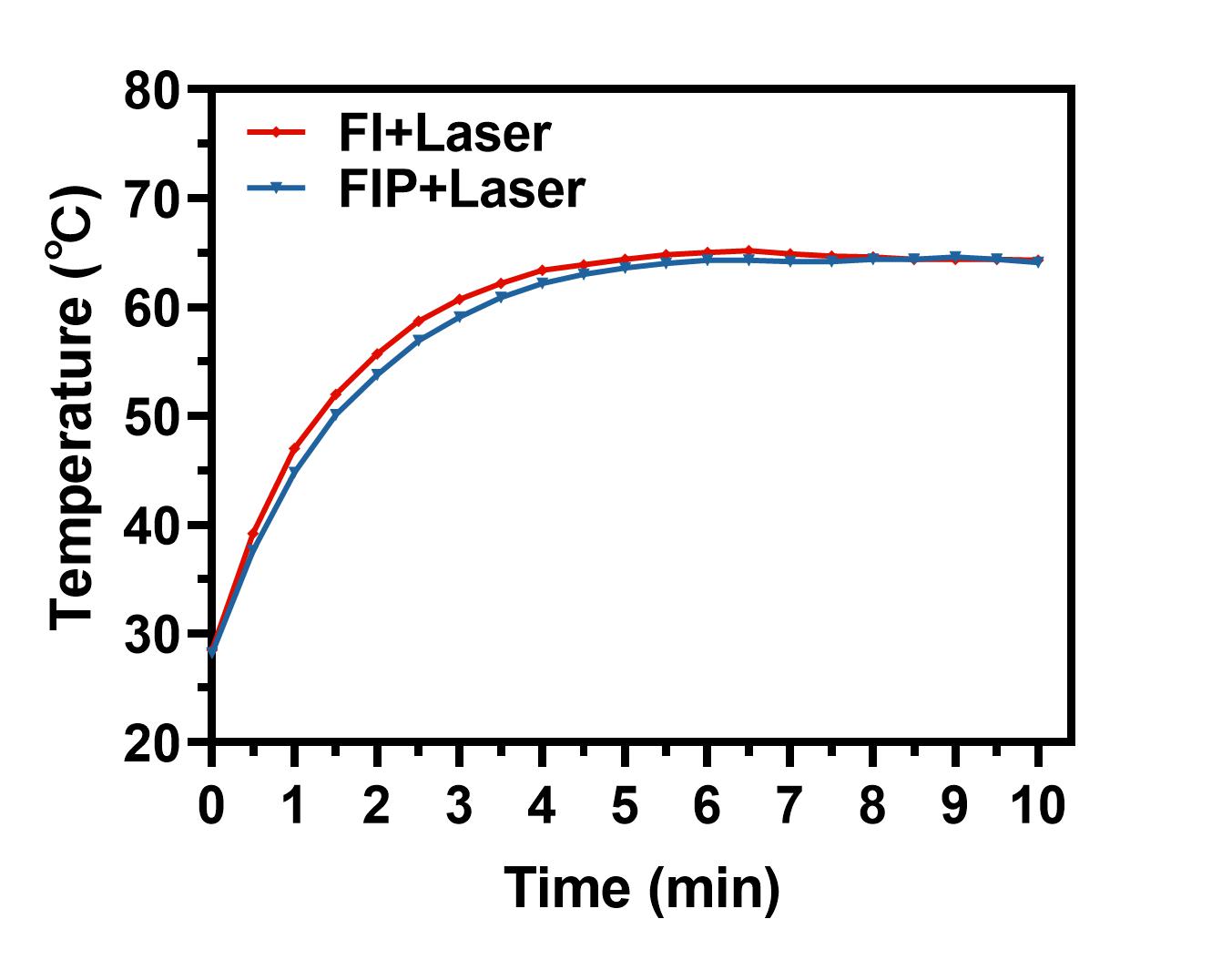


**Figure S10** Temperature change curves of FIP and FI under exposure to the 808 nm laser.

**
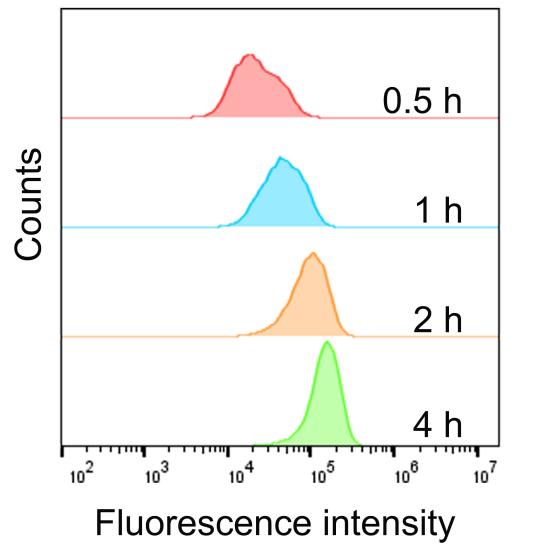
**

**Figure S11** Flow cytometry analysis of intracellular uptake of FIP.

**
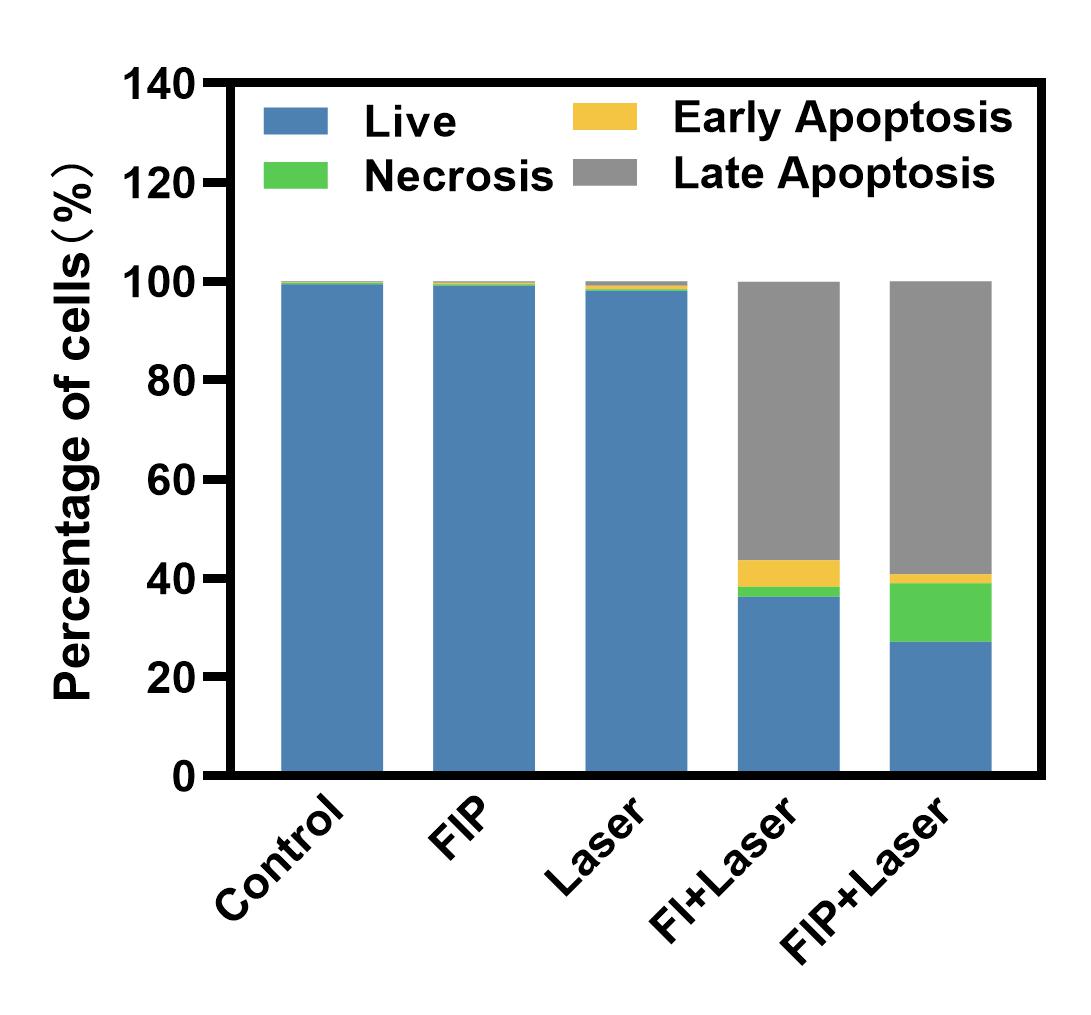
**

**Figure S12** Flow cytometry analysis of the cell apoptosis of 4T1 cells after different treatments.

**
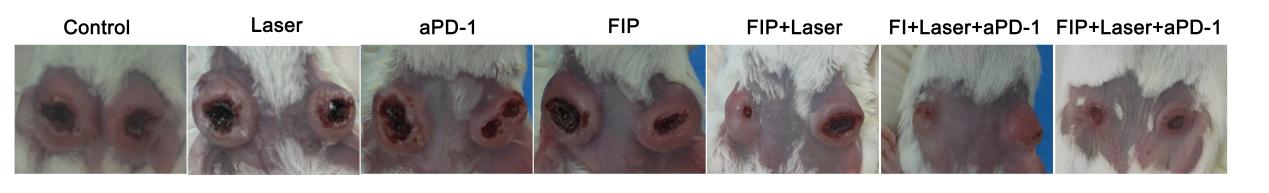
**

**Figure S13** Representative digital photos of 4T1 tumors on both sides of BALB/c mice in different groups on day 16.

**
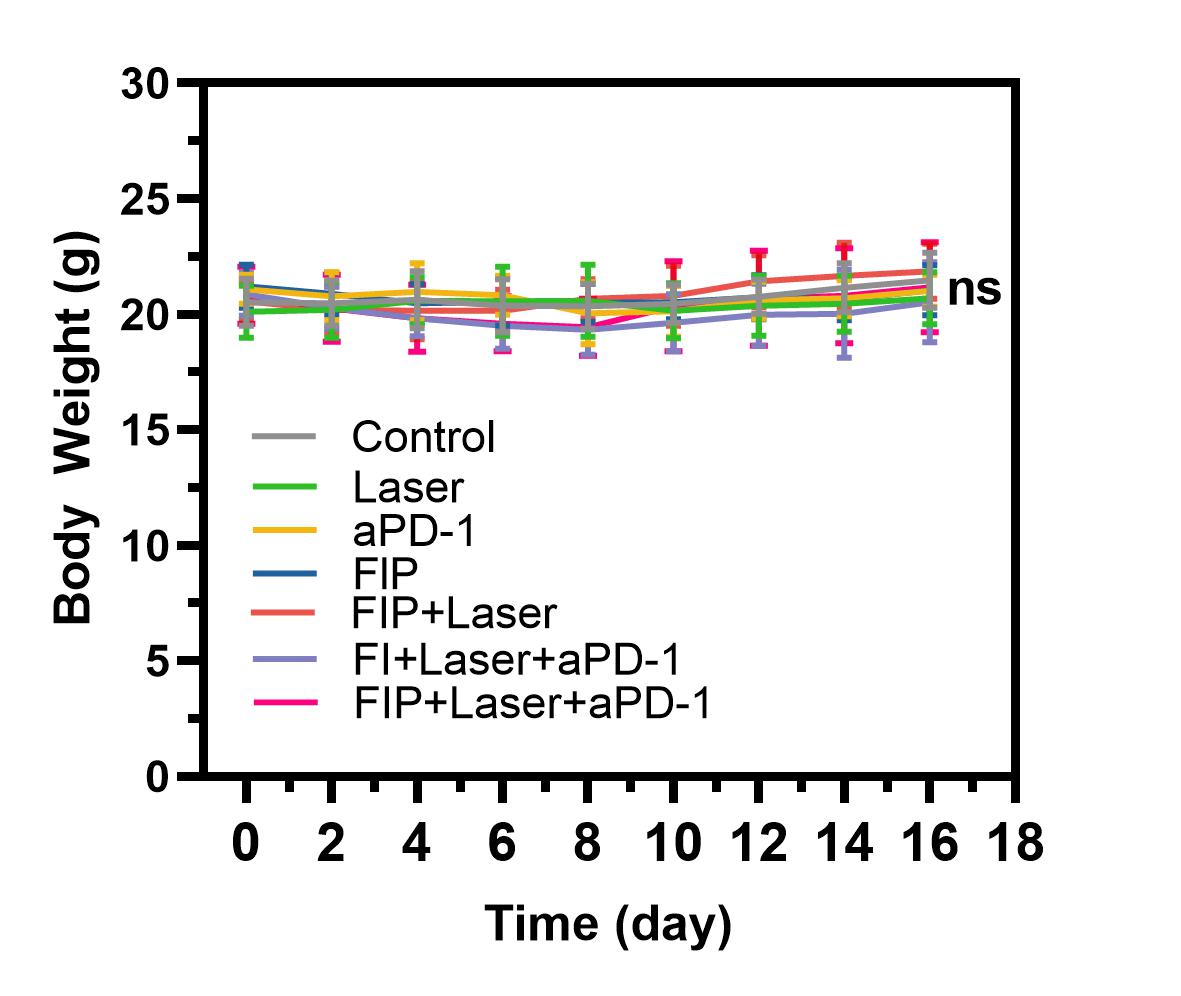
**

**Figure S14** Time-dependent body weight curves of the 4T1 tumor-bearing mice after different treatments.

**
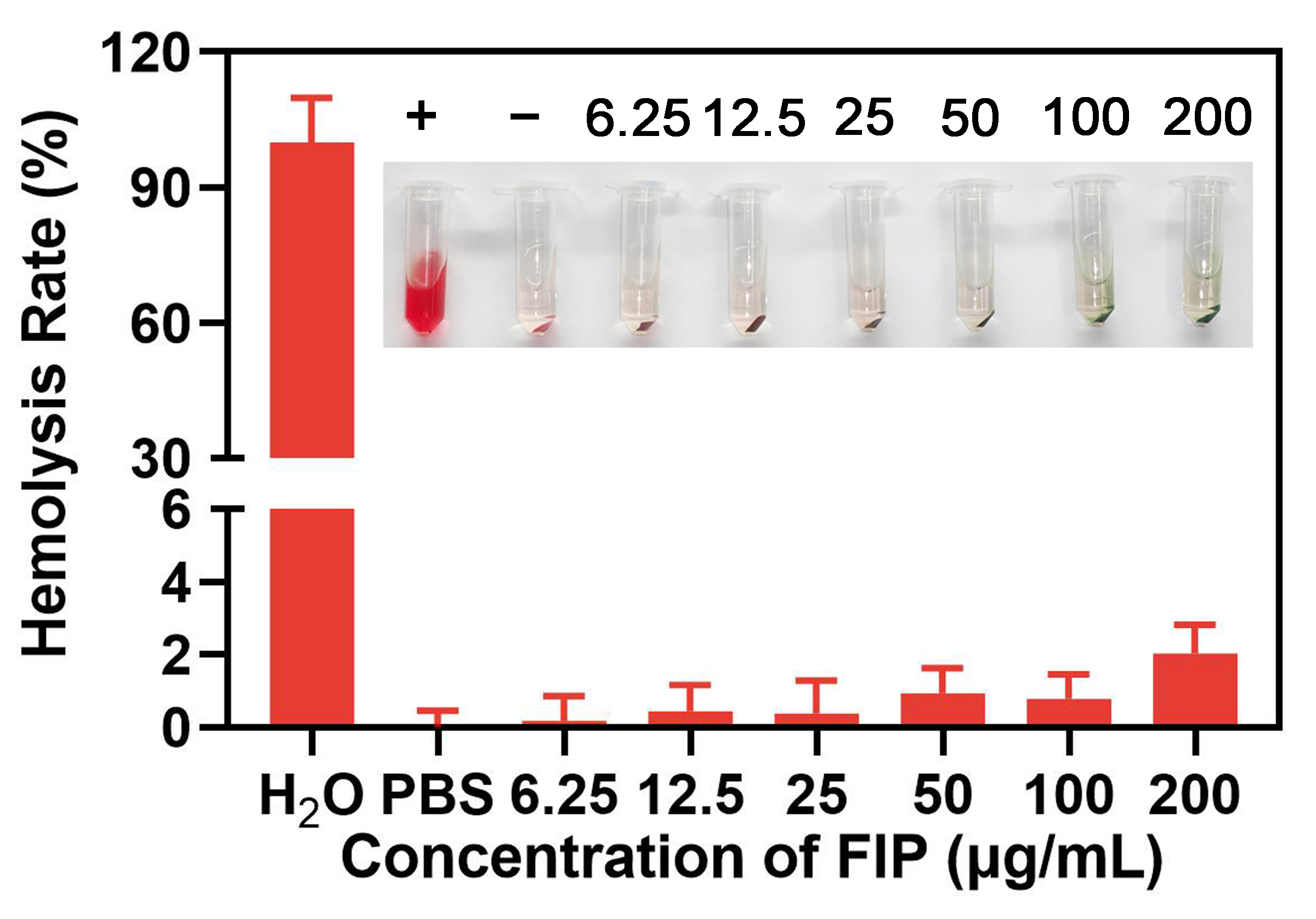
**

**Figure S15** Hemolysis rate of RBCs treated with FIP at various concentrations.

**
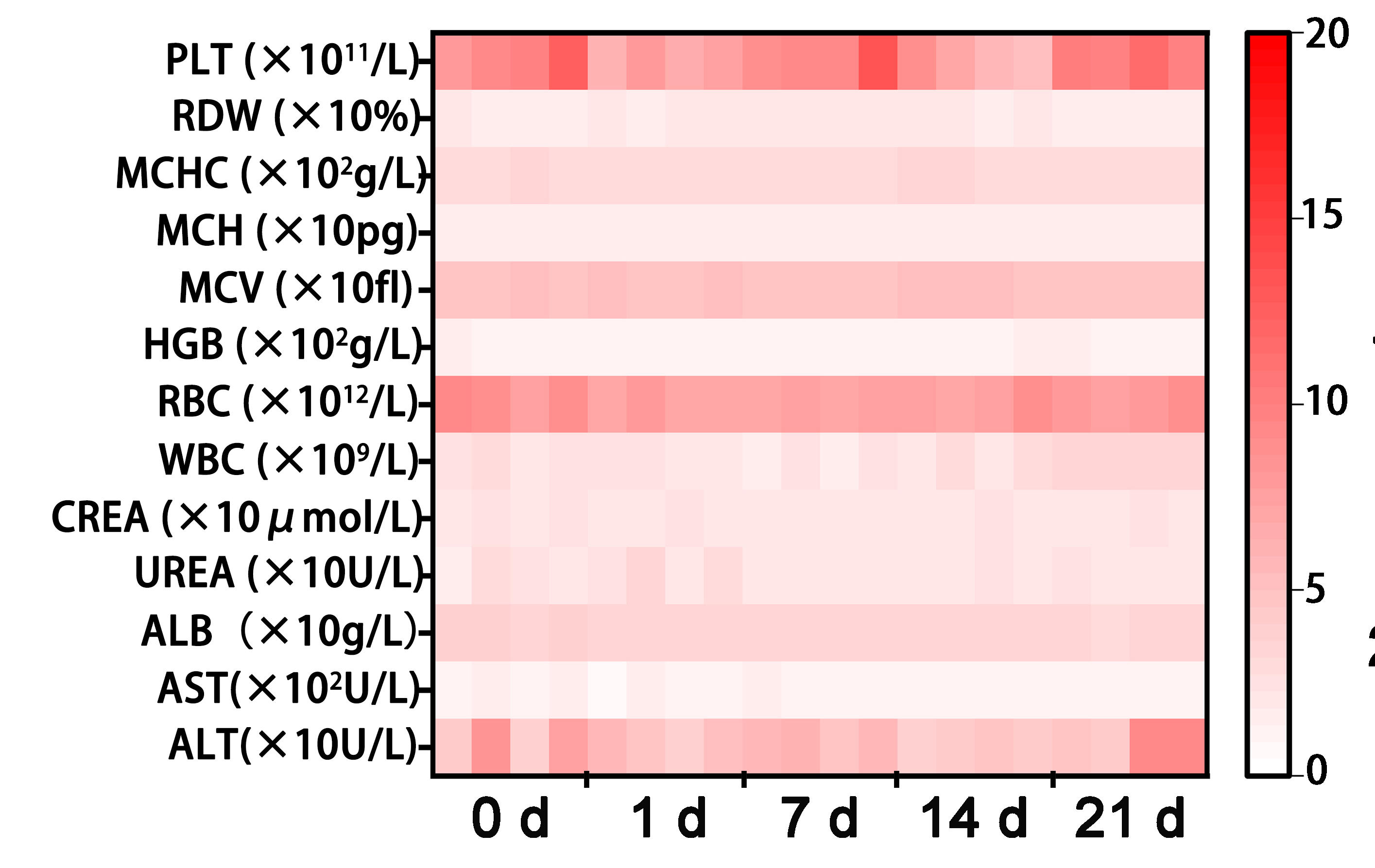
**

**Figure S16** Hematological and blood biochemical tests were performed on mice following intravenous injection of FIP at various times.


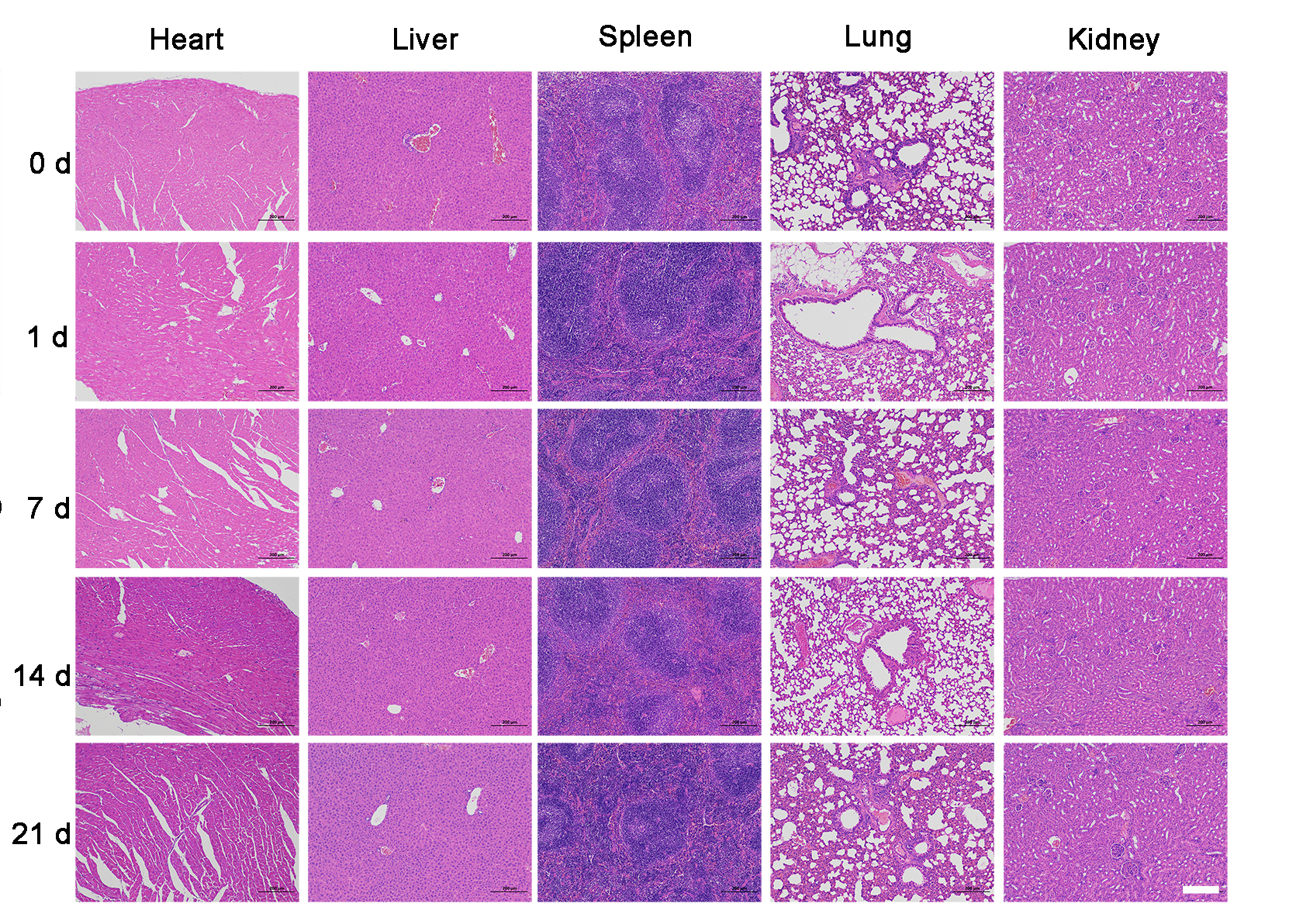


**Figure S17** H&E staining of major organs of mice after different treatments.
